# Secreted Nonstructural Protein 3 is a Pathogenic Determinant of *Orbivirus*

**DOI:** 10.1002/advs.75255

**Published:** 2026-04-13

**Authors:** Junyong Guan, Dong Zhou, Ran Shao, Xing Liu, Jin Peng, Yingran Huang, Shuhui Qi, Cankun Xi, Menghang Wang, Bin Sun, Yinglin Qi, Xin Yin

**Affiliations:** ^1^ State Key Laboratory of Animal Disease Control and Prevention Harbin Veterinary Research Institute Chinese Academy of Agricultural Sciences Harbin China; ^2^ Research Center for Pharmacoinformatics College of Pharmacy Harbin Medical University Harbin China

**Keywords:** hemorrhage, nonstructural protein 3 (NS3), orbivirus, secretion, vascular leakage

## Abstract

*Orbivirus* is a genus of double‐stranded RNA viruses that infect a broad spectrum of arthropods and vertebrates. Hemorrhage across different organs is a hallmark pathological feature of orbivirus infection, as exemplified by the bluetongue virus (BTV); however, the underlying mechanisms for this feature remain unclear. This study unravels a previously unrecognized extracellular role of orbivirus nonstructural protein 3 (NS3), which hijacks the conventional secretion pathway and utilizes phosphatidylinositol (4,5)‐bisphosphate to travel across the plasma membrane, ultimately inducing vascular permeability and leakage. Site‐directed mutagenesis reveals a conserved lysine or arginine residue adjacent to the second transmembrane domain of orbivirus NS3 as being critical for secretion. Notably, a single lysine mutation impairing NS3 secretion in BTV has no effect on viral infection in vitro but abolishes vascular leakage in the spleen and lungs, rendering the virus completely avirulent in AG129 mice. This study reveals the extracellular functions of orbivirus NS3, providing critical novel insights into the molecular mechanisms driving the hemorrhagic pathology characteristic of orbivirus infections.

## Graphical abstract

This study uncovers a conserved PIP2‐dependent secretory pathway of orbivirus NS3 that induces vascular leakage. Pharmacological disruption of PIP2‐NS3 interaction significantly reduces viral pathogenicity and provides protective efficacy in murine models, establishing PIP2‐mediated NS3 secretion as both a key virulence determinant and a promising broad‐spectrum therapeutic target for orbivirus‐induced hemorrhagic diseases.

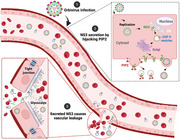

## 1 Introduction

*Orbivirus* is the largest genus within the family *Sedoreoviridae*, comprising at least 22 recognized species. Notable members include bluetongue virus (BTV), epizootic hemorrhagic disease virus (EHDV), African horse sickness virus (AHSV), and equine encephalosis virus (EEV), all of which cause severe clinical symptoms and high mortality in wildlife and domestic animals. Several orbivirus species, such as Corriparta virus, Changuinola virus, Kemerovo virus, and Orungo virus, are zoonotic and capable of infecting humans [1]. Orbiviruses are primarily transmitted among vertebrates through hematophagous arthropods. With rapid climate change, the global incidence and geographical distribution of orbiviruses have increased markedly over the past two decades [2].

The orbivirus genome is composed of ten double‐stranded RNA segments that encode seven structural (VP1–VP7) and five nonstructural (NS1, NS2, NS3/NS3a, NS4, and NS5) proteins [3, 4, 5]. Among these, NS3 is translated from alternative in‐frame start codons, resulting in two isoforms, NS3 and NS3a [6]. As a sole membrane‐associated protein, NS3 is not essential for viral replication, but plays a critical role in virus egress from infected cells, a key function that has been convincingly demonstrated in prior studies by different research groups [7, 8, 9]. NS3 contains two conserved hydrophobic domains that contribute to its viroporin‐like properties and facilitate virus release [10, 11, 12]. The highly conserved PSAP motif in BTV NS3 interacts with TSG101, a component of the ESCRT‐I complex, to mediate the release of this virus. Mutations disrupting the TSG101‐binding motif of BTV NS3 significantly impair virus budding [8, 13]. In addition to its role in viral egress, BTV NS3 activates the MAPK/ERK signaling pathway, a key mediator of apoptosis, to induce cell lysis [14]. It may also interfere with the interferon response to BTV infection in vitro [15]. Notably, NS3/NS3a knockout mutations are safe and confer protection against virulent BTV challenges in sheep, which is suggestive of a role for NS3 in viral virulence in vivo [16]. However, the precise mechanisms through which NS3 contributes to the pathogenesis of these viruses are poorly understood.

Besides producing progeny virions, infected cells actively secrete significant quantities of noninfectious viral components, including flavivirus NS1, rotavirus (RV) NSP4, and severe acute respiratory syndrome coronavirus 2 (SARS‑CoV‑2) ORF8 [17, 18, 19]. These secreted viral proteins perform diverse biological functions, such as suppressing host immune defenses, inducing organ dysfunction [20], and causing tissue damage to facilitate the survival and propagation of virus [21, 22]. Proteins destined for secretion generally follow two pathways: the conventional protein secretion pathway, typically utilized by proteins with an N‐terminal signal sequence, or the unconventional secretion pathway, utilized by proteins lacking a signal peptide. In the conventional pathway, proteins are synthesized in the endoplasmic reticulum (ER) and transported to the Golgi apparatus (GA) via COPII vesicles, where they are modified before being secreted [23]. Conversely, unconventional secretion bypasses the ER‐Golgi route, rendering it resistant to brefeldin A (BFA), a potent modulator of the conventional secretion pathway [23]. Viral proteins exhibit diverse secretory mechanisms. For instance, the hepatitis E virus (HEV) ORF2 protein employs the conventional ER‐Golgi pathway for secretion. In contrast, nonclassical pathways are utilized by the SARS‐CoV‐2 NSP2 and the Ebola virus (EBoV) matrix protein VP40, which bypass the canonical secretory systems [24, 25, 26]. Notably, SARS‐CoV‐2 ORF8 exhibits dual dependency, utilizing both conventional and nonclassical pathways for secretion [19].

Infections caused by orbiviruses, particularly BTV, EHDV, and AHSV, lead to vascular injury and dysfunction, culminating in hemorrhage in various tissues [2]. Immune responses, inflammatory and vasoactive mediators, cytokine storms, and related immunopathological mechanisms collectively play a major role in the developing vascular leakage [27, 28, 29, 30, 31]. However, no key viral proteins that are directly responsible for mediating vascular leakage and contributing to the pathogenesis of orbiviruses have been identified.

This study aimed to explore the contribution of NS3 to the pathogenesis of orbiviruses. The study unraveled the membrane‐associated secretion capability of orbivirus NS3. The secreted NS3 directly triggered endothelial barrier dysfunction in vitro and localized vascular leakage in the dorsal dermis of mice. On the contrary, defective NS3 secretion prevented vascular leakage in the spleen and lungs, leading to significant attenuation of viral pathogenicity in BTV‐infected AG129 mice. This study provides critical mechanistic insights into NS3‐induced pathogenesis of orbivirus infection and presents a potential therapeutic target for hemorrhagic diseases caused by orbiviruses.

## 2 Results

### 2.1 Orbivirus NS3 Proteins Exhibit Conserved Secretory Capacity

**FIGURE 1:**
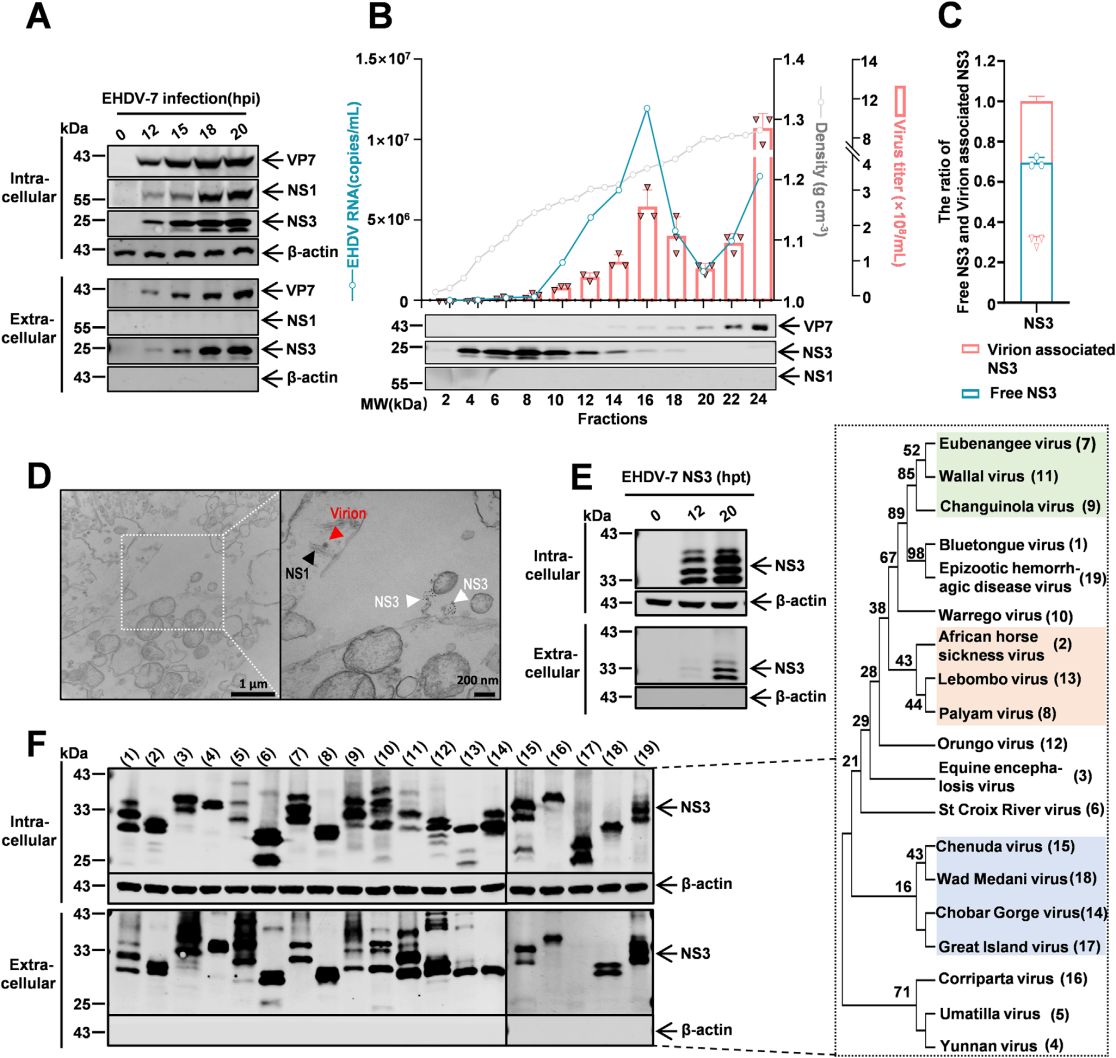
Orbivirus NS3 proteins exhibit conserved secretory capacity. (A) Detection of NS3 release in response to EHDV infection. BHK‐21 cells were infected with EHDV‐7 (multiplicity of infection (MOI) = 5), and both the infected cells and culture medium were harvested at 12, 15, 18, 20 h post‐infection (hpi) for western blotting. (B) Clarification of the association between extracellular NS3 and virus particles. Culture fluids from EHDV‐7‐infected (MOI = 5) BHK‐21 cells at 18 hpi were separated via sucrose density gradient centrifugation. Fractions were collected for EHDV RNA detection, western blotting, TCID_50_ assay, and density determination. (C) Protein levels of free NS3 (fractions 3–8) and virion‐associated NS3 (fractions 9–18) were determined using Image J, and the ratio of two NS3 forms was calculated. (D) Transmission electron micrograph of a thin section of EHDV‐7‐infected BHK‐21 cells immunolabeled with the anti‐NS3 antibody. Virion (red arrow) and gold‐labeled NS3 (white arrows) are indicated. Scale bar = 200 nm. (E) Confirmation of the secretory capacity of NS3 based on transfection. HEK‐293T cells were transfected with plasmids expressing EHDV NS3, and both cells and culture medium were collected at 12 and 20 h post‐transfection (hpt) for western blotting. (F) Detection of the secretion of known orbivirus NS3 proteins. Plasmids expressing NS3 proteins from 19 orbivirus species were transfected into HEK‐293T cells. NS3 secretion was detected via western blotting (F left) and a phylogenetic tree based on the 19 orbivirus NS3 proteins was constructed using MEGA 11 (F right). The data shown in **C** are means ± SD (*n* = 3 biologically independent experiments).

Despite a substantial number of studies indicating crucial roles of orbivirus NS3 during virus infection, the functional characterization of this protein has been limited to its intracellular roles [8, 9, 14, 15]. To explore the potential extracellular role of orbivirus NS3, we investigated its presence in the extracellular space. During the early stages (12–20 h) of infection in BHK‐21 cells, the virus replicated without cell lysis, as evidenced by unchanged plasma membrane permeability to trypan blue (Figure S1A). Notably, while the structural protein VP7 was found in the extracellular space, the nonstructural protein NS3 of BTV and EHDV (but not NS1) was detected in the culture fluids of infected cells (Figure 1A and Figure S1B). The egress of NS3 was also confirmed in BTV‐ and EHDV‐infected MDOK cells (Figure S1C,D), a well‐characterized ovine epithelial cell line that more accurately reflects natural host biology, further indicating the specific release of NS3 into the extracellular space without disturbing the cell membrane integrity. As previously reported [32, 33], NS3 associated with the released virions enclosed within transient lipid or extracellular vesicles (EVs). To clarify the association between extracellular NS3 and viral particles, supernatants from EHDV‐infected BHK‐21 cells were collected at 18 h post‐infection (hpi) and analyzed via sucrose gradient centrifugation. Viral genome quantification and TCID_50_ assays revealed that two populations of virus particles were present in fractions 11–18 and 21–24 (Figure 1B). Unlike the typical orbivirus particles in fractions 21–24, those in fractions 11–18 contained a small amount of NS3 protein, resembling lipid membrane‐enclosed particles (Figure S1E,F) [24]. Nevertheless, a large amount of virion‐unassociated NS3 was predominantly detected in fractions 3–10 (Figure 1C), indicating that NS3 in the extracellular space was released independently of the nonlytic release of transient lipid‐ or EV‐enclosed virions. Immunoelectron microscopy confirmed the presence of NS3 in the extracellular space of EHDV‐infected cells (Figure 1D). Viral secretory proteins, including the human immunodeficiency virus (HIV) Nef protein, are secreted under noninfectious conditions [34]. Consistently, overexpression of NS3 from EHDV‐7 and BTV‐20 in HEK‐293T cells exhibited a time‐dependent secretion pattern (Figure 1E; Figure S1G). These data strongly indicate that BTV and EHDV NS3 are released into the extracellular space via a nonlytic secretory pathway. To determine whether the NS3 proteins of other orbiviruses share this secretory property, NS3 sequences from 17 orbivirus species were analyzed (Figure 1F). Despite relatively low amino acid identity (12.4%–55.6%) (Figure 1F; Figure S2, right), secondary structure predictions revealed a conserved topology across the NS3 proteins (Figure S3). This topology included two hydrophobic transmembrane domains, an extracellular domain, and distinct N‐ and C‐terminal cytoplasmic regions, as previously reported [7, 35]. Detection of extracellular protein via western blotting showed that all NS3 proteins, except that of Great Island virus, were secreted into the supernatant of the transfected cells (Figure 1F). Moreover, approximately 7–10 µg/mL of NS3 was detected in the serum of mice infected with BTV‐20 using a BTV NS3 capture ELISA (Figure S1H), which indicated that NS3 secretion also occurred during orbivirus infection in vivo. Collectively, these findings support the notion that secretion is a conserved feature of orbivirus NS3 proteins, underscoring their potential functional significance in the viral life cycle.

### 2.2 Secreted NS3 is Associated With Extracellular Vesicles

**FIGURE 2:**
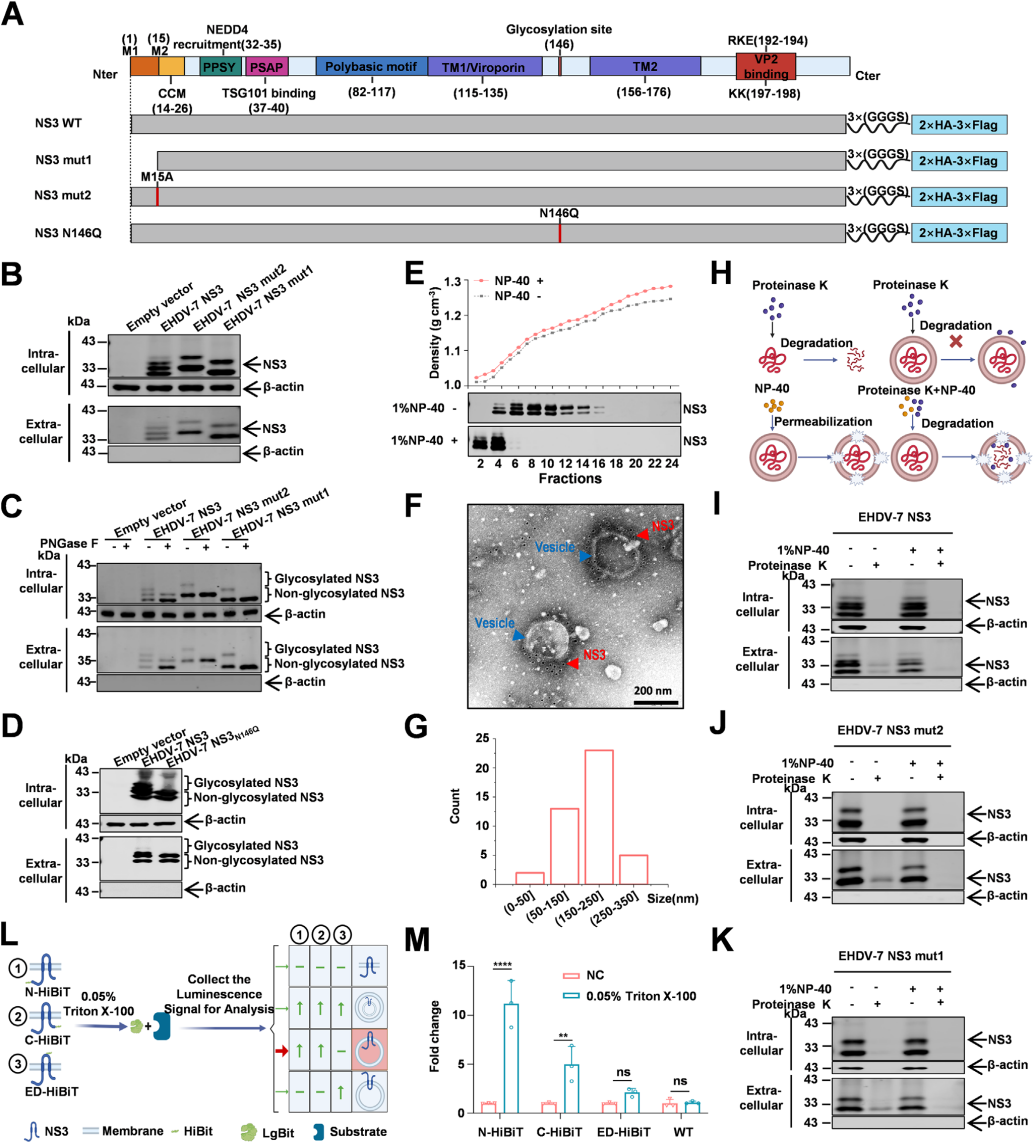
Secreted NS3 is associated with extracellular vesicles. (A) Schematic representation of the main functional domains of EHDV NS3 and strategies for constructing wild‐type (WT) and mutant proteins. EHDV NS3 contains two functional methionines, M1 (AA1) and M2 (AA15). NS3 mut1 is translated from the second methionine (M2, AA15), whereas NS3 mut2 carries an M15A mutation deleting the second methionine, and NS3_N146Q_ carries an N146Q mutation abolishing glycosylation. All forms of NS3 were fused with a flexible linker (GGGS × 3) and 2 × HA‐3 × Flag tag, increasing the molecular weight of NS3 to 32–34 kDa. (B) Characterization of the secreted NS3 isoform. Empty vector (negative control), EHDV NS3 WT, mut1, and mut2 were transfected into HEK‐293T cells. The cells and culture medium were collected at 20 hpt for detecting the secreted protein via western blotting. (C) PNGase F was used to confirm the glycosylation status of secreted NS3. Both cells and culture medium samples from EHDV NS3 WT‐, mut1‐, and mut2‐transfected HEK‐293T cells were treated with PNGase F or PBS (negative control) and analyzed via western blotting. The glycosylated and non‐glycosylated NS3 are indicated in the images. (D) EHDV NS3_N146Q_ was used to assess whether glycosylation is indispensable for NS3 secretion. Both cells and culture medium samples from EHDV NS3 WT and EHDV NS3_N146Q_ were collected at 20 hpt for detection of secreted protein via western blotting. (E) Verification of the association between membrane and secreted NS3 using NP‐40 treatment. Culture medium from EHDV NS3‐transfected HEK‐293T cells was collected at 20 hpt, then treated with 1% NP‐40 (NP‐40+) or left untreated (NP‐40−), and separated via sucrose density gradient centrifugation. Fractions were collected for density determination and western blotting. (F) Immunoelectron micrograph of EHDV NS3 from fraction 7; vesicles (turquoise blue arrow) and gold‐labeled NS3 (red arrow) are indicated. (G) The size of NS3‐associated vesicles was quantified using ImageJ (parentheses denote exclusion and brackets denote inclusion). A total of 43 NS3‐positive vesicles were counted. (H–K) Proteinase K and NP‐40 treatments were used to assess whether secreted NS3 is membrane‐coated. Samples from EHDV NS3 WT‐ (I), NS3 mut2‐ (J), and NS3 mut1 (K)‐transfected HEK‐293T cells were treated with 200 µg/mL proteinase K and 1% NP‐40, and then analyzed via western blotting. (L,M) Mapping the membrane topology of secreted NS3 via Nano‐Glo HiBiT Extracellular Detection Assay. HiBiT tags were fused to the N‐terminus (N‐HiBiT), C‐terminus (C‐HiBiT), and extracellular domain (ED‐HiBiT) of EHDV NS3. Culture medium was collected at 20 hpt, treated with 0.05% Triton X‐100, and analyzed for luminescence signals. PBS treatment served as a negative control. The data shown in M are means ± SD (n = 3 biologically independent experiments). Statistical analysis was performed using two‐way ANOVA, followed by Dunnett's post hoc test for multiple comparisons. ^*^ *p* < 0.05, ^**^ *p* < 0.01, ^***^ *p* < 0.001, ^****^ *p* < 0.0001; ns, not significant.

To characterize the properties of the secreted NS3, constructs encoding the full‐length (NS3, mut2) or truncated (NS3a, mut1) isoforms of EHDV/BTV NS3 were transfected into HEK‐293T cells (Figure 2A,B). Both isoforms were detected in supernatants (Figure 2B; Figure S4A), which was indicative of their ability to be secreted. PNGase F treatment revealed that the secreted NS3 exists in both glycosylated and non‐glycosylated forms (Figure 2C; Figure S4B). Furthermore, the removal of the glycosylation sites did not affect the secretion of NS3 (Figure 2D), indicating that glycosylation is not an essential modification required for NS3 secretion. Sucrose gradient centrifugation of supernatants from EHDV NS3‐transfected cells revealed that NS3 was predominantly localized in fractions 3–10, consistent with observations in EHDV‐infected cells. After nonionic detergent NP‐40‐induced destruction of the membrane components, NS3 secretion was detected in fractions 1–4 (Figure 2E), indicating the lipid membrane‐association of the secreted NS3. Furthermore, immunoelectron microscopy of fraction 7 confirmed that NS3 was primarily associated with vesicles ranging in diameter from 50 to 250 nm (Figure 2F,G). To further evaluate whether NS3 was membrane‐bound or fully enveloped, extracellular NS3 was treated with proteinase K in the presence or absence of NP‐40 (Figure 2H). Most of the NS3 was degraded by proteinase K, even in the absence of NP‐40, whereas NP‐40 treatment rendered all NS3 susceptible, indicating that NS3 was not entirely enclosed by the lipid membrane and had exposed regions under native conditions (Figure 2I–K; Figure S4C–E). The topology of secreted NS3 was further examined using a HiBiT tagging system with tags fused to the N‐terminal, C‐terminal, or extracellular domain (ED) of EHDV NS3 (Figure 2L). Upon treatment with 0.05% Triton X‐100, a significant increase in luminescence was observed for both the N‐ and C‐terminal‐tagged NS3, indicating that these domains reside within the vesicular lumen (Figure 2M). Collectively, these findings indicate that secreted NS3 is associated with extracellular vesicles, with its topology suggestive of partial (extracellular domain) exposure to the extracellular environment.

### 2.3 Conventional Secretory Pathway is Involved in NS3 Secretion

**FIGURE 3:**
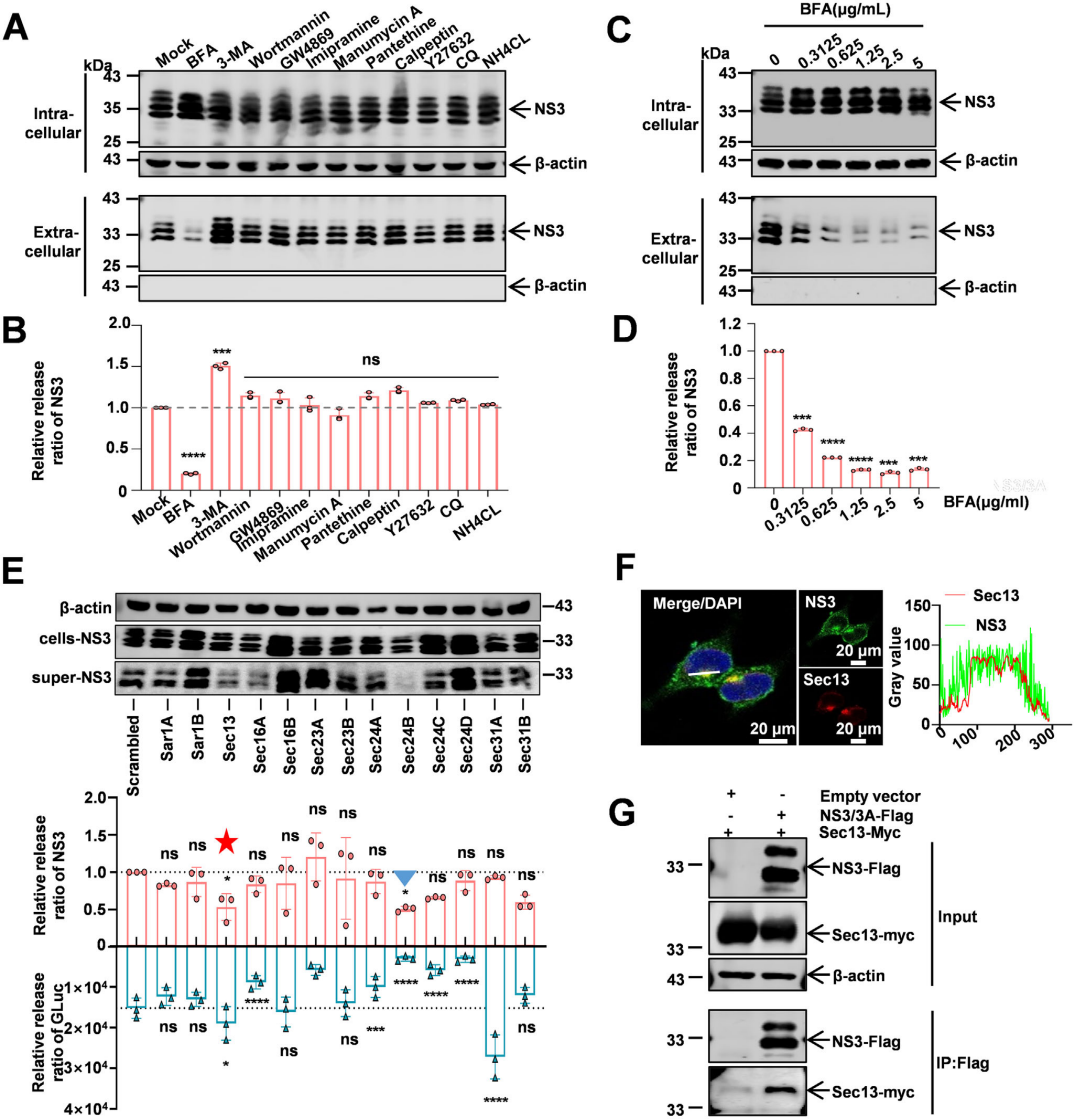
Conventional secretory pathway is involved in NS3 secretion. (A) Pharmacological screening of EHDV NS3 secretion pathways using inhibitors: 3 µg/mL brefeldin A (BFA), 10 mm 3‐methyladenine (3‐MA), 500 nm wortmannin, 5 µm GW4869, 500 nm imipramine, 250 nm manumycin A, 20 µm pantetheine, 2 µm calpeptin, 5 µm Y‐27632, 20 µm chloroquine (CQ), and 20 µm NH_4_Cl. HEK‐293T cells transfected with EHDV NS3 plasmids were treated with inhibitors at 6 hpt, with cells and medium harvested at 20 hpt for western blotting analysis. (B) Quantification of (A) by ImageJ, showing the effects of inhibitors on EHDV NS3 secretion normalized to mock‐treated controls (set as 1). (C) Dose‐response analysis of BFA (0.3125–5 µg/mL) in EHDV NS3‐expressing HEK‐293T cells, assessed by western blotting. (D) ImageJ quantification of (C) showing BFA effects on secretion in a dose‐ dependent manner. (E) RNAi screening of Sec proteins in HEK‐293T cells transfected with either EHDV NS3 WT or Gaussia luciferase (Gluc). The siRNA sequences for the target genes were shown in Table S1. The secretion levels of EHDV NS3 were detected by western blotting, and luciferase activity was measured via Gaussia luciferase assay. Hits reducing both NS3 and Gluc secretion are indicated with a turquoise blue inverted triangle, while those specifically decreasing NS3 secretion are marked with a red pentagram. (F) Confocal microscopy of BHK‐21 cells co‐transfected with EHDV NS3 WT and Sec13, fixed at 20 hpt, with fluorescence quantified using Image J. (G) Co‐immunoprecipitation (Co‐IP) demonstrating EHDV NS3‐Sec13 interaction. The data shown in B, D, E are means ± SD (*n* = 3 biologically independent experiments). Statistical analysis was performed using one‐way ANOVA, followed by Dunnett's post hoc test for multiple comparisons. ^*^ *p* < 0.05, ^**^ *p* < 0.01, ^***^ *p* < 0.001, ^****^ *p* < 0.0001; ns, not significant.

Orbivirus NS3 exerts cytotoxic effects by disrupting the membrane integrity in a time‐dependent manner [10, 11, 12, 36]. To rule out the possibility that early stage NS3 secretion was caused by increased cell membrane permeability or damage, both EHDV NS3 and eGFP were overexpressed in HEK‐293T cells. Notably, NS3 was detected in the supernatant as early as 20 hpt (Figure S5A), a time point when cell membrane integrity was still intact according to trypan blue exclusion and LDH release assays, whereas membrane compromise was not observed until 36 and 48 hpt (Figure S5B,C). These findings confirmed that although NS3 has the reported cytotoxic capacity, early stage NS3 secretion occurs via a mechanism independent of its cytotoxic effects. We employed a panel of inhibitors targeting the cargo trafficking pathways to uncover the mechanism underlying NS3 secretion. Among them, brefeldin A (BFA) significantly suppressed NS3 secretion in a dose‐dependent manner (Figure 3A–D), implicating the conventional secretion pathway, which is dependent on COPII‐coated vesicles for intracellular protein trafficking. Furthermore, siRNAs targeting 13 key Sec proteins that constitute COPII vesicles were transfected into HEK‐293T cells, followed by overexpression of EHDV NS3 or Gaussia luciferase, a well‐established marker of the conventional secretory pathway. qPCR results validated the efficient knockdown of all the targeted Sec proteins (Figure S5D). With the depletion of Sec13 and Sec24B, the secretion of NS3 was significantly reduced, indicating a pivotal role of COPII vesicles in NS3 secretion. Sec13 depletion specifically reduced NS3 secretion without affecting the secretion of Gaussia luciferase (Figure 3E). Furthermore, confocal microscopy revealed the colocalization of NS3 and Sec13 (Figure 3F), and coimmunoprecipitation (Co‐IP) assays confirmed a specific interaction between NS3 and Sec13 under both transfection and infection conditions (Figure 3G; Figure S5E,F). These findings highlight the specific role of Sec13 in mediating NS3 secretion via a conventional secretory pathway.

### 2.4 NS3 Hijacks Phosphatidylinositol 4,5‐bisphosphate to Travel Across the Plasma Membrane Barrier

**FIGURE 4:**
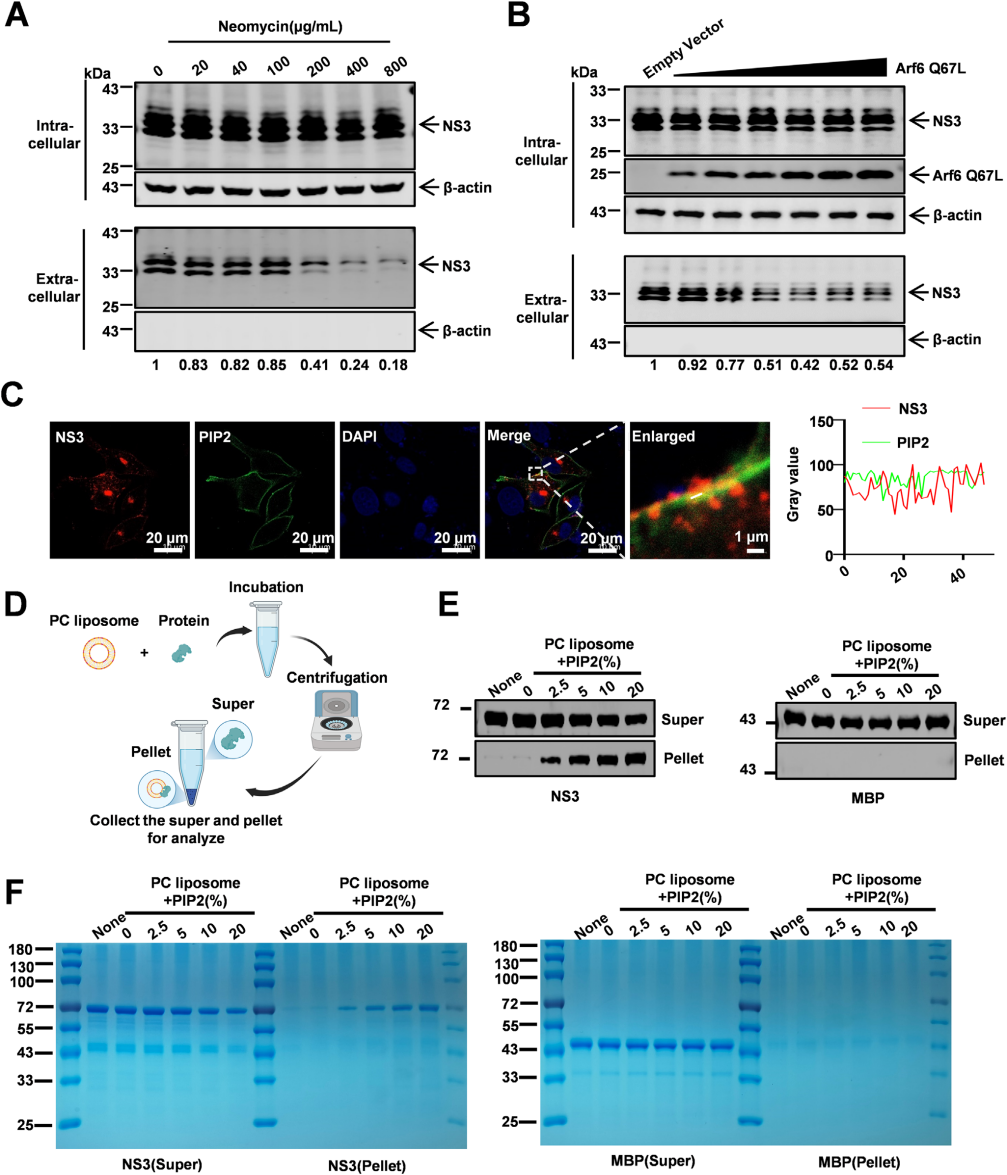
NS3 hijacks phosphatidylinositol 4,5‐bisphosphate (PIP2) to traverse the plasma membrane barrier. (A) Dose‐dependent effects of neomycin (20–800 µg/mL), a PIP2‐binding inhibitor, on EHDV NS3 secretion. HEK‐293T cells transfected with EHDV NS3 plasmids were treated with neomycin at 6 hpt, with cells and medium harvested at 20 hpt. Secreted NS3 levels (relative to 0 µm control) were analyzed by western blotting and quantified using ImageJ (values below blots). (B) Secretion modulation by Arf6 Q67L (0.25–1.5 µg) co‐transfected with 1.5 µg EHDV NS3 in HEK‐293T cells, assessed by western blotting and quantified as in (A). (C) Subcellular localization of EHDV NS3 WT and PH‐PLCδ‐GFP (PIP2 probe) in BHK‐21 cells fixed at 20 hpt, imaged by confocal microscopy and quantified with ImageJ. (D–F) Liposome co‐sedimentation assay for NS3‐PIP2 interaction: (D) Schematic of PC liposome preparation; (E) western blotting and (F) SDS‐PAGE analysis of supernatants (S) and pellets (P) after incubating NS3 with PC liposomes containing 2.5%–20% PIP2.

Although NS3 utilizes the conventional secretion pathway for transport, its two conserved hydrophobic domains compel it to span and localize to the cell membrane, suggesting that NS3 interacts with specific molecules in the plasma membrane to facilitate secretion. Phosphatidylinositol 4,5‐bisphosphate (PIP2), a critical plasma membrane lipid, facilitates the translocation of various viral proteins, including HIV Tat and EBoV VP40 [37, 38] and interacts with BTV NS3 and VP5 [39]. To explore the role of PIP2 in NS3 secretion, neomycin, a PIP2‐binding inhibitor that specifically sequesters PIP2 by masking its interaction domains [37, 40, 41, 42], was applied to NS3‐expressing cells. This treatment resulted in a dose‐dependent inhibition of NS3 secretion (Figure 4A; Figure S6A). Additionally, the overexpression of Arf6 Q67L, a constitutively active Arf6 mutant that removes PIP2 from the plasma membrane [43], significantly inhibited NS3 secretion in a dose‐dependent manner (Figure 4B). Confocal microscopy further corroborated this finding, revealing the colocalization of NS3 and PIP2 at the cell membrane (Figure 4C). To further verify the interaction between NS3 and PIP2, recombinant NS3 protein was purified to >95% purity, as verified using SDS‐PAGE analysis, from the supernatant of transiently transfected HEK‐293F cells (Figure S6B,C). Subsequent multilamellar vesicles containing different ratios of phosphatidylcholine (PC) and PIP2 were prepared using the extrusion method and confirmed via electron microscopy (Figure S6D,E) [44]. A liposome sedimentation assay was conducted using the purified NS3 protein in combination with PIP2‐containing liposomes to assess their interactions (Figure 4D). As the ratio of PIP2 in the PC liposomes increased, the amount of NS3 co‐sedimented with the liposomes also increased in a dose‐dependent manner (Figure 4E,F). Collectively, these findings strongly support the hypothesis that NS3 interacts with PIP2 to travel across the plasma membrane.

### 2.5 Highly Conserved K/R Residue Adjacent to the Second Transmembrane Domain is Critical for NS3 Secretion

**FIGURE 5:**
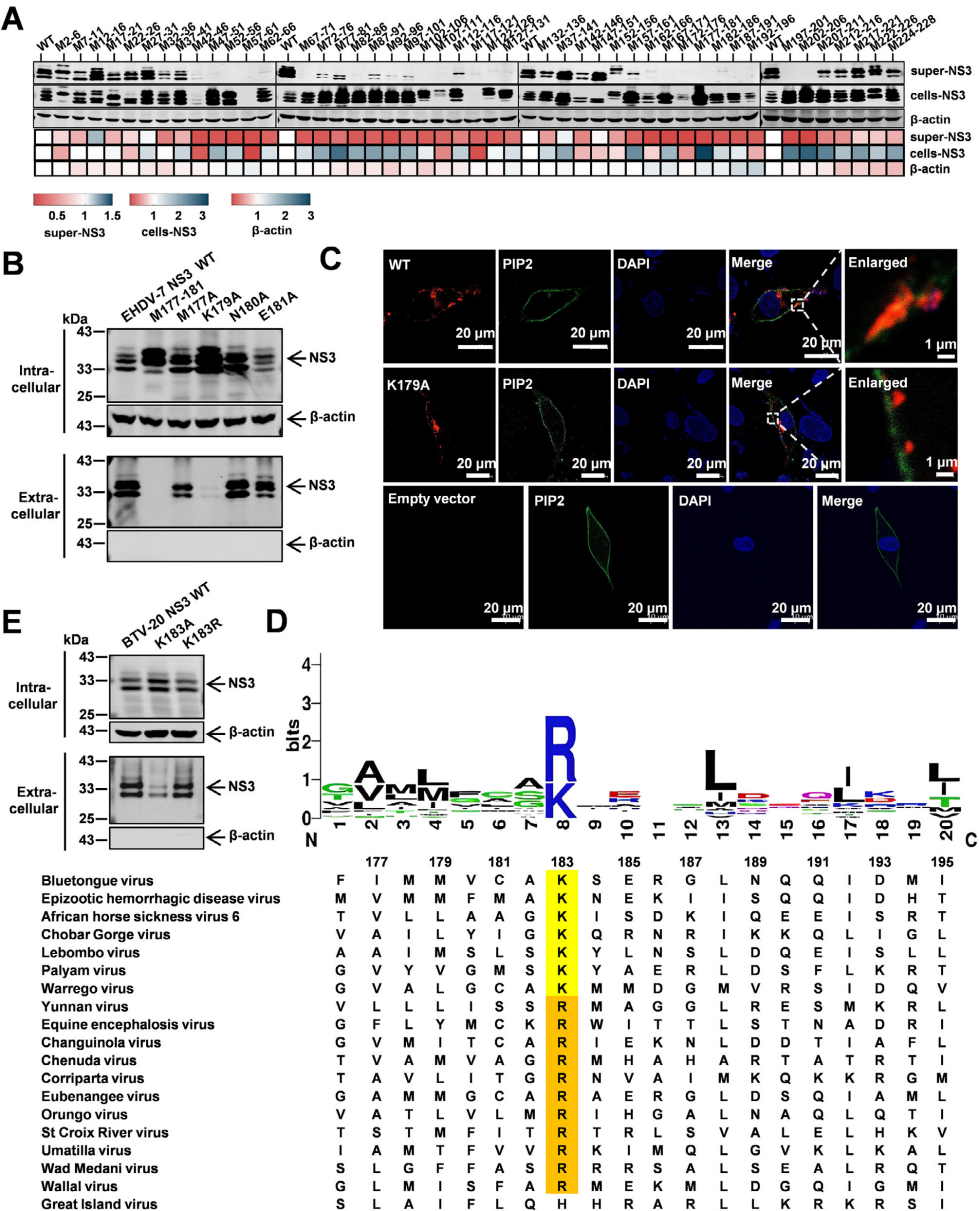
The highly conserved K/R residues adjacent to the second transmembrane domain are critical for NS3 secretion. (A) Secretory profiles of EHDV NS3 mutants in HEK‐293T cells analyzed by western blotting. Supernatant NS3 (super‐NS3), cellular NS3 (cells‐NS3), and β‐actin levels were quantified using ImageJ and normalized to NS3 WT, presented as a heatmap (mean ± SD, *n* = 3). “M” denotes 5‐alanine substitutions at indicated positions. (B) Comparative secretion of EHDV NS3 WT and single‐site mutants assessed by western blotting. (C) PIP2 localization in BHK‐21 cells co‐transfected with PH‐PLCδ‐GFP (PIP2 probe) and either EHDV NS3 WT, EHDV NS3_K179A_, or empty vector, fixed at 20 hpt and imaged by confocal microscopy. (D) Conserved motif prediction for orbivirus NS3 proteins using MEME based on NS3 sequence alignments. (E) Functional validation of lysine/arginine residues in NS3 secretion: HEK‐293T cells transfected with wild‐type BTV NS3 (WT), BTV NS3_K183A_, or BTV NS3_K183R_ were analyzed at 20 hpt by western blotting of cell lysates and supernatants.

To identify the residues essential for EHDV NS3 secretion, a 5‐alanine (A) scanning mutagenesis was performed. Analysis of overexpressed NS3 mutants revealed that all NS3 mutants with five consecutive alanine substitutions targeting two discrete regions (AA 42–131 and 162–206) exhibited significant secretory impairment, without affecting cell viability or membrane integrity (Figure 5A; Figure S7A,B). Notably, neither of the mutations in the PSAP motif of NS3, which is critical for the release of virus, nor the knockdown of TSG101, a binding partner of the PSAP domain, affected NS3 secretion (Figure S7C,D), indicating that NS3‐mediated virus release and its secretion are different processes that use distinct functional domains. Although numerous regions of NS3 affect its secretion, mutations at sites involved in the regulation of secretion and other reported functions would complicate the phenotypic interpretation. Therefore, this study focused on structural elements with functional specificity and secretion‐modulating potential, specifically key residues that significantly impair NS3 secretion without disrupting its documented biological activities. Identification of such residues will establish a molecular basis for constructing “secretion‐deficient yet functionally intact” NS3 mutant, enabling precise dissection of NS3 secretion in virus infection. In particular, mutations in the AA 177–181 region, which is adjacent to the second transmembrane domain and has no reported function, resulted in increased intracellular NS3 levels and reduced extracellular NS3 levels (Figure 5A). Further site‐directed mutagenesis revealed a mutation (K179A) at lysine (K) 179 in EHDV NS3, which nearly abolished NS3 secretion (Figure 5B). Confocal microscopy confirmed that the K179A mutation did not alter the colocalization of EHDV‐7 NS3 with the ER or Golgi apparatus (Figure S7E,F) or its interaction with PIP2 (Figure S7G). However, whereas WT NS3 localized to the outer side of PIP2 at the plasma membrane, the K179A mutant was confined to the inner side of PIP2 (Figure 5C). This indicates that the K179A mutation specifically disrupts the ability of NS3 to travel across the plasma membrane barrier.

Conserved motif prediction using MEME (MEME Suite 5.5.7) revealed that the K or arginine (R) adjacent to the second transmembrane domain is conserved across most orbivirus NS3 proteins, with the exception of the Great Island virus NS3 (Figure 5D). Site‐directed mutagenesis of BTV NS3 confirmed that the K183A mutation significantly inhibited secretion, whereas the K183R mutation did not (Figure 5E). To define the essential role of the K/R residue in NS3 secretion, we performed all‐atom molecular dynamics (MD) simulations of WT and K183A mutant NS3 in a POPC bilayer. The K183A mutant exhibited greater stability (lower RMSD, Figure S8A,B) and embedded more deeply than the WT, as measured by the protein‐lipid center of mass distance (Figure S8C,D). We attribute this to the loss of hydrogen bonds between residue 183 and lipid headgroups in the mutant (Figure S8E). We propose that in the WT NS3, these bonds act as an anchor, preventing deeper insertion and facilitating secretion, while their loss in the K183A mutant promotes deeper embedding and membrane retention. Collectively, these findings indicate that the lysine or arginine residue adjacent to the second transmembrane domain is essential for the secretion of orbivirus NS3, underscoring its indispensable role in secretion.

### 2.6 Orbivirus NS3 Triggers Endothelial Permeability and Vascular Leakage

**FIGURE 6:**
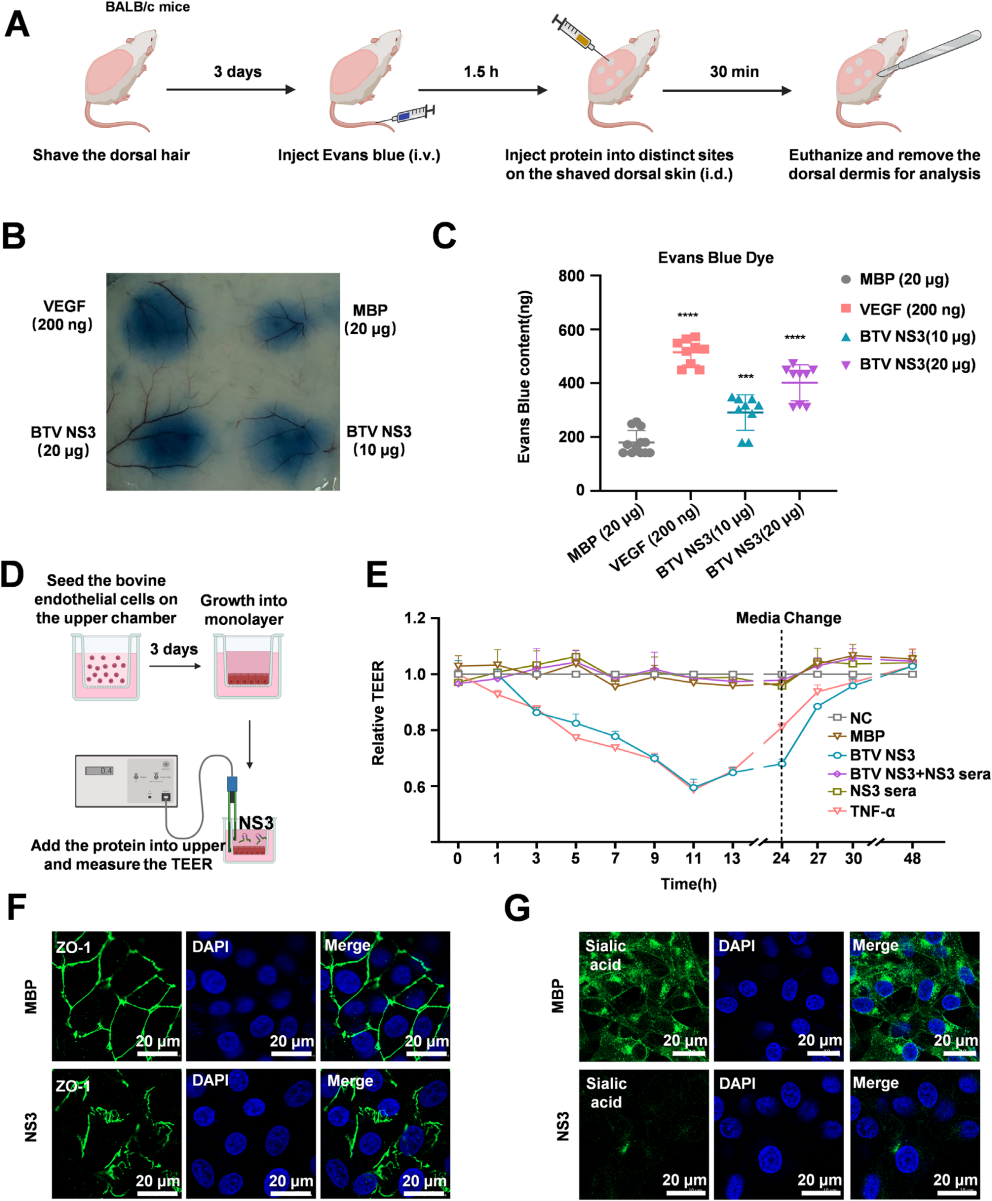
Orbivirus NS3 triggers endothelial permeability and vascular leakage. (A) Schematic of the Evans Blue dermal Miles assay. (B) Representative image showing vascular leakage in mouse dorsal dermis after Evans Blue injection, with VEGF (positive control) and MBP (negative control). (C) Quantification of Evans Blue extravasation: Leakage sites from (B) were excised with a surgical scalpel, and dye was extracted for absorbance measurement at 630 nm. (D) Schematic of the trans endothelial electrical resistance (TEER) measurement system. (E) TEER‐based assessment of bovine endothelial barrier disruption by secreted NS3 proteins, with TNF‐α (positive control) and MBP (negative control). (F,G) Confocal microscopy of bovine endothelial monolayers showing ZO‐1 distribution (F) and sialic acid localization (G) after treatment with purified BTV NS3, using MBP‐treated cells as negative controls. The data shown in C are means ± SD from nine independent biological replicates (*n* = 9 animals). Statistical analysis was performed using one‐way ANOVA, followed by Dunnett's post hoc test for multiple comparisons. ^*^ *p* < 0.05, ^**^ *p* < 0.01, ^***^ *p* < 0.001, ^****^ *p* < 0.0001; ns, not significant.

The fact that NS3 knockout mutation in BTV leads to avirulence in sheep [16], prompted us to investigate the role of NS3 in vascular permeability, a key factor underlying the hemorrhagic symptoms of orbivirus infections. The Evans Blue dermal Miles assay was performed to evaluate the direct effects of NS3 on vascular hyperpermeability (Figure 6A). Evans blue dye was intravenously injected into the blood circulation system of mice, and was followed by intradermal injection of distinct proteins on the shaved dorsal skin. The vascular injury caused by proteins was reflected by the leakage of Evans blue. VEGF (positive control), purified BTV NS3, and EHDV NS3 induced significant leakage of the Evans Blue dye, which exceeded the levels observed for maltose‐binding protein (MBP; negative control) (Figure 6B,C; Figure S9A), indicating that the secreted NS3 is capable of inducing vascular leakage. Next, we used a trans endothelial electrical resistance (TEER) assay to assess the effect of NS3 on endothelial permeability (Figure 6D). Bovine endothelial cells were used to form a confluent monolayer and establish the TEER, a key indicator of barrier integrity. Treatments that induce hyperpermeability in the monolayer lead to a reduction in TEER, reflecting a compromised barrier function. NS3 treatment markedly increased the endothelial hyperpermeability, an effect that was significantly mitigated by anti‐NS3 sera but not by the negative control serum (Figure 6E; Figure S9B), which indicated its specific role in increasing the endothelial hyperpermeability. To investigate whether NS3 enhances endothelial permeability via canonical signaling cascades, we assessed the effects of pharmacological inhibitors targeting Src kinase (PP2) and Rho GTPase–ROCK (Y‐27632) pathways. Using TEER assays, we first confirmed that both inhibitors effectively attenuated VEGF‐induced endothelial permeability, validating their functional activity in our experimental system (Figure S9C). However, subsequent TEER measurements revealed that neither PP2 nor Y‐27632 alleviated NS3‐induced barrier disruption (Figure S9D). Notably, NS3 treatment could disrupt the integrity of tight junctions, as evidenced by the loss of ZO‐1 staining in the bovine endothelial cell monolayers (Figure 6F; Figure S9E). Moreover, NS3 treatment significantly disrupted sialic acid, a key component of the endothelial glycocalyx (Figure 6G; Figure S9F). Collectively, these findings indicate that orbivirus NS3 increases the endothelial permeability, leading to vascular leakage, and highlight its potential role in driving the hemorrhagic manifestations of orbivirus infection.

### 2.7 Secreted NS3 is a Pivotal Determinant of Viral Pathogenicity

**FIGURE 7:**
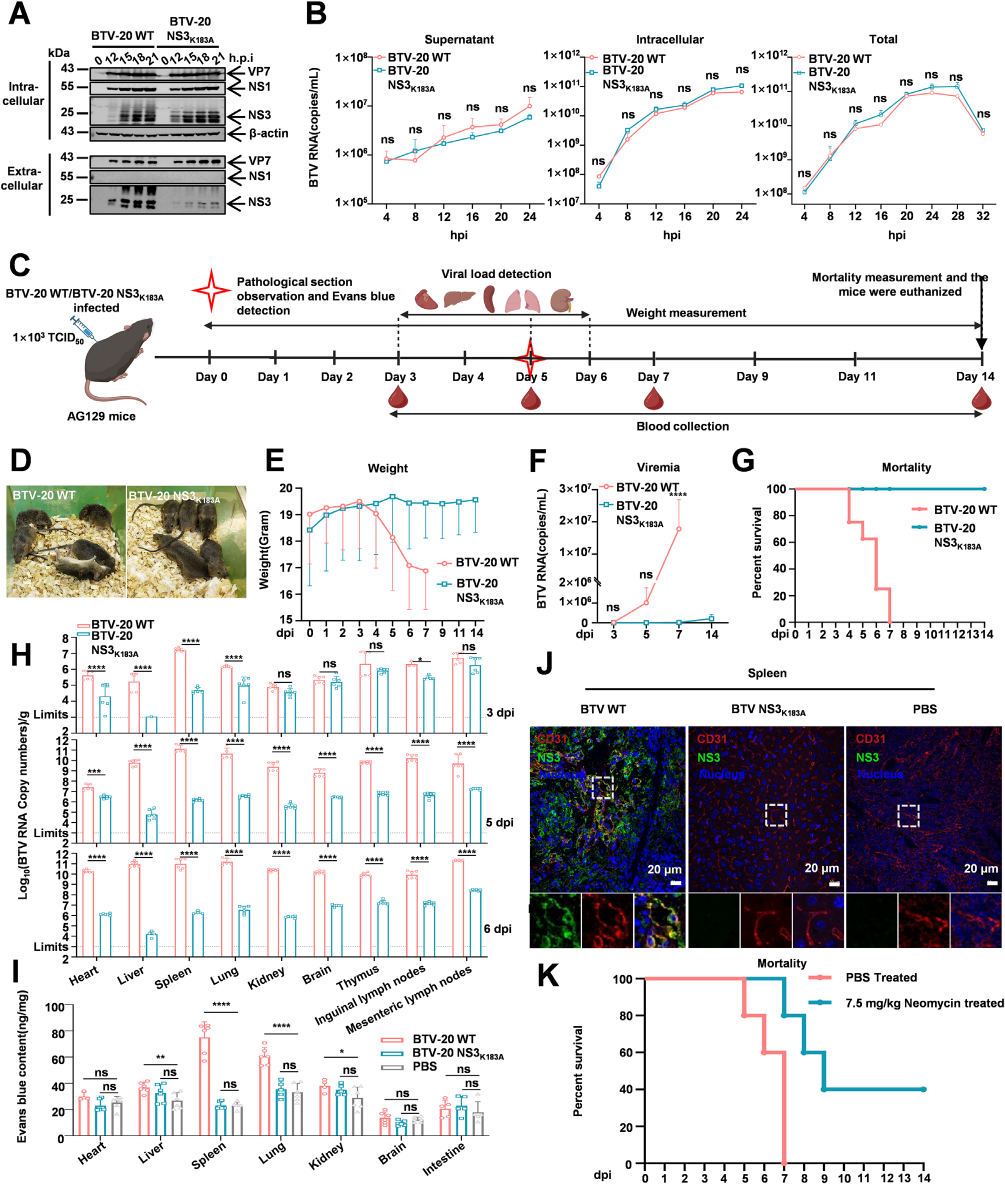
Secreted NS3 is a pivotal determinant of viral pathogenicity. (A) Validation of BTV NS3_K183A_ secretory deficiency: BHK‐21 cells infected with BTV WT or BTV NS3_K183A_ (MOI = 5) were harvested at 12–20 h post‐infection (hpi), with NS3 secretion analyzed by western blotting. (B) Replication kinetics of BTV WT versus BTV NS3_K183A_ in BHK‐21 cells, quantifying viral RNA in extracellular, intracellular, and total fractions. (C) Experimental design: AG129 mice were subcutaneously (s.c.) infected with 1 × 10^3^ TCID_50_ of BTV WT or BTV NS3_K183A_. (D) Representative images of infected mice at 4 days post‐infection (dpi). (E) Weight monitoring of infected mice (*n* = 5). (F) Viremia quantification by qRT‐PCR (*n* = 5). (G) Survival analysis over 14 days post‐infection (*n* = 8). (H) Tissue viral loads measured by qPCR at 3–6 dpi (*n* = 6). (I) Vascular permeability assessment via Evans Blue extravasation in tissues at 5 dpi (*n* = 6). (J) Immunofluorescence of spleen sections showing NS3 (green) and CD31^+^ vascular structures (red). (K) Therapeutic evaluation: AG129 mice infected with BTV WT (1×10^3^ TCID_50_, s.c.) received daily i.v. injections of neomycin (7.5 mg/kg/day) or PBS (vehicle control), with survival monitored for 14 days (*n* = 5). The data shown in B, E‐F, H‐I are means ± SD. Statistical analysis was performed using two‐way ANOVA, followed by Dunnett's post hoc test for multiple comparisons. ^*^ *p* < 0.05, ^**^ *p* < 0.01, ^***^ *p* < 0.001, ^****^ *p* < 0.0001; ns, not significant.

To elucidate the role of secreted NS3 in viral pathogenesis in vivo, we generated BTV NS3_K183A_, a secretion‐deficient BTV mutant, based on the BTV strain BTV‐20/GX015/China/2013, which has well‐established animal models [45]. In vitro analysis revealed that the NS3 levels in the supernatant of BHK‐21 cells and MDOK cells infected with BTV S10_K183A_ were significantly reduced compared with those of wild‐type (WT) BTV, whereas VP7 and viral RNA levels remained comparable (Figure 7A,B; Figure S10A). This indicated that the K183A mutation specifically disrupts NS3 secretion without affecting the overall viral replication cycle in vitro.

AG129 mice were infected with the WT or BTV NS3_K183A_ (Figure 7C). Mice infected with WT BTV exhibited severe symptoms, including lethargy, piloerection, significant weight loss, high‐titer viremia, and 100% mortality within 7 dpi. In contrast, BTV NS3_K183A_‐infected mice remained asymptomatic, showed no weight loss, did not develop viremia, and exhibited no mortality, even at 14 dpi (Figure 7D–G). These results strongly support the pivotal role of secreted NS3 in determining viral pathogenicity. Given that AG129 mice lack interferon signaling, we further evaluated the role of secreted NS3 in immunocompetent hosts. In wild‐type BALB/c suckling mice, intracerebral inoculation with BTV‐20 WT resulted in 44.4% mortality by 14 dpi, whereas no deaths were observed in mice infected with BTV‐20 NS3_K183A_ (Figure S10B). We also assessed viral pathogenicity in Merino sheep, a natural host of BTV. Sheep infected with BTV‐20 WT developed pronounced fever and high‐titer viremia between 3 and 7 dpi. In contrast, sheep infected with BTV‐20 NS3_K183A_ exhibited only mild, transient fever with low‐level viremia detectable briefly at 4–5 dpi, accompanied by substantially reduced clinical signs (Figure S10C,D). All these findings further demonstrate that NS3 secretion is critical for orbivirus pathogenicity in immunocompetent and natural hosts.

Viral load analysis showed significantly higher viral loads in the heart, liver, spleen, lungs, and lymph nodes of WT‐infected mice than in those of BTV NS3_K183A_‐infected mice, particularly at 5 dpi and beyond (Figure 7H). Histopathological analysis revealed extensive tissue damage, including splenic necrosis, edema, alveolar thickening, hepatocyte degeneration, thymic disruption, and lymph node necrosis, in BTV WT‐infected mice. In contrast, BTV NS3_K183A_‐infected mice displayed no significant pathology, similar to the PBS control mice (Figure S10E). This further indicates that NS3 secretion was sufficient to render the virus completely avirulent in AG129 mice. The Evans Blue dye assay confirmed significant vascular leakage in the spleen and lungs of BTV WT‐infected mice, but not in BTV NS3_K183A_‐infected mice (Figure 7I), indicating loss of the secretory capacity rendering the virus unable to induce vascular leakage in tissue, a main reason for hemorrhagic manifestations. This may explain why BTV NS3_K183A_ was completely avirulent in AG129 mice. Transcriptome analysis revealed significant alterations in genes associated with the tight junction and VEGF signaling pathways in the spleen and lungs following infection with WT BTV (Figure S11), which further supports the role of secreted NS3 in driving vascular leakage. Notably, colocalization of NS3 and CD31, a vascular marker, was observed in the spleen of WT BTV‐infected mice (Figure 7J), providing a functional model wherein secreted NS3 directly interacts with the vasculature to induce vascular leakage.

Given that NS3 is a secreted viral protein that plays a critical role in pathogenesis in vivo, we next attempted to clarify the secreted NS3 in the sera of AG129 mice infected by NS3 immunity. AG129 mice were immunized with either MBP (negative control) or purified NS3 twice at 14‐day intervals, followed by a challenge with WT BTV (Figure S12A). NS3‐immunized mice developed robust neutralizing antibody titers, with significantly enhanced responses following booster immunization (Figure S12B). MBP‐immunized mice exhibited typical severe symptoms, including lethargy, piloerection, and mortality by day 8, whereas NS3‐immunized mice showed milder symptoms, delayed mortality, and one survivor beyond 14 dpi, although viremia was observed in both groups (Figure S12C–E). Building on the protective effects of NS3 immunization, we evaluated whether pharmacological targeting of NS3 secretion could mitigate BTV pathogenesis (Figure S13A). BTV‐infected AG129 mice were treated with neomycin, a PIP2‐targeting inhibitor that blocks NS3 secretion in vitro (Figure 4A; Figure S6A). The neomycin‐treated mice exhibited markedly attenuated clinical symptoms, significantly lower viremia, and delayed disease progression, with a 40% survival rate at 14 dpi. This contrasted sharply with the PBS‐treated controls, which uniformly succumbed to infection by day 7 (Figure 7K; Figure S13B,C). These findings indicate that secreted NS3 is essential for inducing vascular leakage and plays a critical role in orbivirus pathogenesis. Thus, NS3 is a promising target for the treatment of orbivirus‐induced diseases.

## 3 Discussion

Secreted viral proteins play pivotal roles in the viral life cycle and pathogenesis by modulating the host environment to facilitate infection and immune evasion. These proteins can directly manipulate cellular processes, for example, by altering the extracellular matrix, disrupting intercellular junctions, and modulating signaling pathways to promote viral dissemination [46, 47, 48]. Additionally, they often act as key regulators of host immune responses by interfering with cytokine signaling [19, 21]. Beyond these functional roles, some secreted viral proteins serve as decoys to neutralize neutralizing antibodies or create favorable niches for viral replication [21, 26]. Their ability to influence host–pathogen interactions underscores their importance in determining viral virulence and disease progression, making them attractive targets for therapeutic interventions and vaccine development. The NS3 protein of orbiviruses has been well‐characterized for its crucial role in mediating the release of virus particles. However, the current understanding of the functions of orbivirus NS3 is largely confined to its intracellular activity. In this study, we identified the NS3 protein encoded by orbiviruses as a secreted pathogenic determinant, challenging the previous understanding of NS3 as an exclusively intracellular protein required for viral egress. Observations as early as 1991 revealed the presence of BTV NS3 in the extracellular spaces of BTV‐infected cells, where it was hypothesized to facilitate virion budding or extrusion from the plasma membrane [33]. Recent studies have shown that NS3 can be associated with the virions contained in transient lipid or EVs and is released via nonlytic mechanisms [32]. Our findings extend this knowledge by demonstrating that NS3 is present in the supernatant of infected cells before cell membrane rupture due to viral infection, with at least 70% of the secreted NS3 existing independently of the infectious virions. Notably, alterations in motifs, such as PSAP and PPSY, linked to NS3‐mediated viral egress, did not affect NS3 secretion. Knockdown of TSG101, a host factor that interacts with the PSAP motif, had no discernible effect on NS3 secretion. These observations indicate that the PSAP–TSG101 interaction, which is important for other NS3‐associated functions such as viral release, is not essential for NS3 secretion. They establish a foundational framework for exploring novel extracellular functions of secreted NS3, independent of its role in conventional viral egress mechanisms.

Although orbivirus NS3 lacks a signal peptide, the two conserved transmembrane (TM) domains present in all NS3 proteins facilitate recognition by the host secretory machinery, directing it either to the classical secretory pathway or to the Type *IV* nonclassical pathway. The hallmark of the Type *IV* pathway is its bypassing of the Golgi apparatus, as evidenced by BFA insensitivity and the absence of Golgi‐mediated complex N‐glycosylation [49]. However, we found that NS3 secretion is significantly inhibited by BFA, and that some of the secreted NS3 is glycosylated. These results confirm that NS3 preferentially utilizes the conventional secretory pathway. Furthermore, our data indicate that NS3 secretion requires COPII vesicles, with Sec13 playing a critical role in this process. Sec13, a component of the outer layer of COPII vesicles, is particularly important for the secretion of large TM proteins [50]. The sensitivity of COPII vesicle stability to these proteins highlights the specific role of Sec13 in NS3 secretion. Notably, although NS3 undergoes glycosylation during transit through the Golgi apparatus, the absence of glycosylation does not impair its secretion. This suggests that glycosylation is not essential for NS3 secretion, but is rather a byproduct of its passage through the classical secretory pathway.

Proteins with signal sequences typically enter the ER and are processed and trafficked through the Golgi apparatus to the plasma membrane, where they are directly secreted. However, orbivirus NS3 lacks classical signal sequences but contains two TM domains that enable its insertion into the plasma membrane [35]. Thus, a membrane‐associated molecule is probably required to assist NS3 in traversing the membrane barrier and entering the extracellular space. Phosphoinositides, including PIP2, are critical for various cellular trafficking processes, such as actin cytoskeleton remodeling, intracellular signaling, and membrane and cell adhesion [51]. These versatile lipids are frequently exploited by viral and cellular proteins to facilitate their secretion. For example, viral proteins, such as HIV Tat, EBoV VP40, and HIV p17, use PIP2 to anchor to the plasma membrane before being secreted into the extracellular space [37, 38, 52]. Similarly, our findings revealed that orbivirus NS3 exploits PIP2 to mediate its secretion. Interference with PIP2 function significantly inhibited NS3 secretion, and both confocal microscopy and liposome sedimentation assays confirmed the colocalization and interaction between NS3 and PIP2. Notably, NS3 utilizes PIP2 in a distinct manner compared with other viral proteins, such as HIV Tat, EBoV VP40, and HIV p17. Although these proteins primarily employ PIP2 for plasma membrane anchoring and self‐recruitment, NS3 first reaches the plasma membrane via the conventional secretory pathway. Once localized to the membrane, NS3 exploits PIP2 to mediate its translocation across the membrane barrier into the extracellular space. This unique mechanism is consistent with our observation that secreted NS3 associates with vesicles and maintains the inward orientation of its N‐ and C‐termini. Furthermore, this process differs fundamentally from conventional viral secretion mechanisms, such as that for hepatitis E virus (HEV) ORF2, which relies on vesicle‐membrane fusion to release encapsulated proteins. These findings collectively underscore the unique strategy employed by orbivirus NS3 to exploit both classical secretion pathways and host lipid signaling systems for its secretion. Notably, repurposing neomycin—an approved aminoglycoside antibiotic clinically used solely for bacterial infections—as a PIP2‐targeting agent provided 40% protection against lethal BTV challenge in mice. This is the first demonstration of an established antimicrobial drug exhibiting measurable antiviral efficacy, revealing that PIP2 modulation is a promising therapeutic avenue against orbivirus infections.

Mutational analyses revealed domains critical for NS3 secretion, including conserved hydrophobic TM regions essential for ER membrane insertion and polybasic motifs that facilitate membrane binding [53]. However, most of these domains are essential for proper trafficking of NS3 to the cell membrane, a process critical for its functions, including directing VP5 to the membrane for virus maturation, efficient virus production, and release [53, 54]. Using single‐residue mapping within these critical regions, a conserved K/R residue, located immediately downstream of the second TM domain, was identified as essential for NS3 secretion without impacting its trafficking to the plasma membrane. K/R residues are positively charged at physiological pH, rendering them ideal for interacting with negatively charged molecules such as PIP2. However, our liposome sedimentation assays showed that mutation had no effect on interaction with PIP2. Instead, the mutation prevented NS3 from crossing the plasma membrane and trapping it on the inner side of PIP2 as was confirmed via confocal microscopy. All‐atom MD simulations revealed that the K183A NS3 mutant is more stable and embeds more deeply into a POPC membrane than the WT protein. The mutant's lower RMSD and smaller protein‐lipid distance indicate enhanced stability and deeper insertion. This might be caused by the loss of hydrogen bonds between residue 183 and lipid headgroups. We propose that the hydrogen bonds of WT NS3 act as a barrier to deep embedding, explaining why it is more readily secreted than the retained K183A mutant. However, the precise mechanism through which this conserved K/R residue regulates NS3 secretion requires further investigation.

During viral infections, certain virulence factors are released into the extracellular space where they contribute significantly to disease pathogenesis. For example, DENV NS1, a secreted nonstructural protein, induces vascular leakage, playing a central role in dengue hemorrhagic fever and shock syndrome, with its secretion levels correlating with disease severity [20, 55, 56]. Similarly, rotavirus NSP4, secreted via a nonclassical pathway, functions as an enterotoxin, whereas norovirus NS1 suppresses intestinal IFN‐λ responses, exacerbating pathogenesis [18, 21]. Previous studies on orbiviruses, such as BTV, EEV, and ASHV, have identified VP2 and NS3 as critical virulence factors, although their direct roles in vascular leakage remain unclear [11, 57]. Orbivirus infections are often characterized by hemorrhage and vesicular system dysfunction, which are typically attributed to endothelial cell death and cytokine storms. However, our study demonstrates that orbivirus NS3 directly induces vascular leakage. Using the Evans Blue Dermal Miles assay, we showed that NS3 disrupts endothelial integrity by increasing permeability through tight junction and glycocalyx disruption. Furthermore, BTV NS3_K183A_, a secretion‐deficient BTV mutant, which maintains normal replication and release, but lacks NS3 secretion, exhibited drastically reduced pathogenicity in vivo. The infection of mice with the secretion‐deficient mutant resulted in no viremia, significantly reduced viral loads in all tissues, and complete survival. Crucially, these mice were also protected from vascular leakage in key organs like the spleen and lungs, where secreted NS3 was found localized on vesicle surfaces. Immunization against NS3 delayed mortality and provided partial protection, underscoring the protein's pivotal role in pathogenesis. The critical role of NS3 secretion was also validated in natural ruminant hosts, where the mutant virus induced significantly attenuated clinical signs compared to wild‐type BTV. It should be noted that due to ethical considerations and funding constraints, the sample size for the Merino sheep experiment was relatively small (*n* = 3 per group), and the observation period was limited to two weeks post‐infection. Future studies with larger sheep cohorts and extended observation periods will help to further quantify the protective effect and assess potential long‐term outcomes associated with NS3‐mediated pathogenicity.

## 4 Conclusion

**FIGURE 8:**
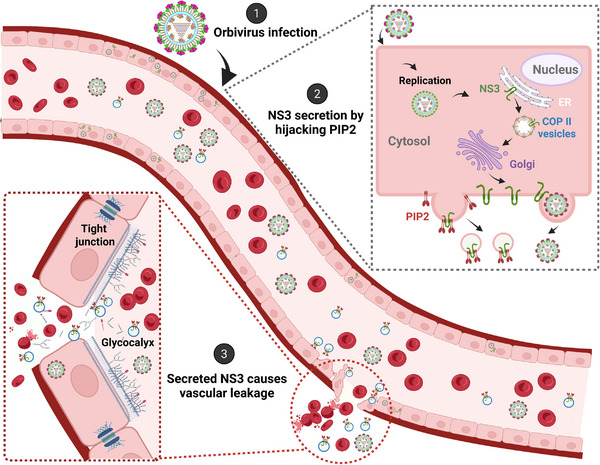
Schematic diagram of the molecular mechanism underlying secreted NS3 protein‐induced vascular leakage in orbivirus infection. After infection of vascular endothelial cells, orbivirus replicates rapidly with production of NS3; the newly synthesized NS3 hijacks COPII vesicles to travel to the plasma membrane, where NS3 inserts, which is followed by budding depending on the assistance of PIP2. Once released from infected vascular endothelial cells, the secreted membrane‐associated NS3 circulates through the blood vascular system, adheres to vessel luminal surfaces, directly disrupts the endothelial glycocalyx, and compromises the tight junction integrity, ultimately leading to vascular leakage.

In conclusion, our study identifies the self‐secretion of orbivirus NS3 as a previously unrecognized and pivotal determinant of viral pathogenesis. We demonstrate that NS3 secretion, facilitated by PIP2, disrupts the endothelial barrier by compromising tight junctions and the glycocalyx, leading to vascular leakage—a hallmark of disease (Figure 8). Critically, inhibiting NS3 secretion with a deficient mutant attenuated virulence, preventing viremia, vascular leakage, and mortality in mice. Collectively, this study elucidates the mechanism of orbivirus‐induced vascular damage and positions secreted NS3 as a key therapeutic target. Strategies aimed at blocking its secretion or neutralizing the extracellular protein offer promising avenues for mitigating orbiviral diseases.

## 5 Experimental Section

### 5.1 Animals, Cells, and Viruses

AG129 mice, which lack both type I and II interferon (IFN) receptors, were kindly provided by Professor Jian‐ping Zuo from the Institute Pasteur of Shanghai, Chinese Academy of Sciences. BALB/c mice were purchased from Liaoning Changsheng Biotechnology Co., Ltd. Merino sheep were purchased from Jilin Qian'an Zhihua Breeding Co., Ltd. All animals were bred under specific pathogen‐free (SPF) conditions at Harbin Veterinary Research Institute, Chinese Academy of Agricultural Sciences, and all animal experiments were approved by the Committee on the Ethics of Animal Experiments of the Harbin Veterinary Research Institute, Chinese Academy of Agricultural Sciences (approval numbers: 240507‐06‐GR for AG129 mice, 250717‐04‐GR for BALB/c mice, and 251031‐01‐GR for Merino sheep).

BHK‐21 (baby hamster kidney cells, ATCC CCL‐10, USA), BSR‐T7/5 (a BHK‐21‐derived clone stably expressing T7 RNA polymerase), HEK‐293T (human embryonic kidney cells, ATCC CRL‐3216, USA), CPAE (bovine pulmonary artery endothelial cells, ATCC, CCL‐209, USA), and MDOK (Madin‐Darby ovine kidney cells, ATCC, CRL‐1633, USA) were maintained in high‐glucose Dulbecco's Modified Eagle's Medium (DMEM, Gibco) supplemented with 8% heat‐inactivated fetal bovine serum (FBS, F0193‐500ML, Sigma), 2 mm L‐glutamine, 100 U/mL penicillin, 100 µg/mL streptomycin, and nonessential amino acids. All cell cultures were maintained at 37°C in a humidified incubator with 5% CO_2_.

The BTV‐20 strain GX015/China/2013 and EHDV‐7 strain YN09‐04 were initially isolated by our laboratory [45, 58]. A secretion‐deficient mutant (BTV NS3_K183A_) was generated by introducing a K183A substitution into the BTV‐20 strain via plasmid‐based reverse genetics [59, 60]. Briefly, full‐length cDNA clones of all ten BTV‐20 genomic segments were cloned into pCI vector (Promega), flanked by T7 promoter and hepatitis delta virus ribozyme sequences. Equimolar plasmid mixtures (1 µg per construct) were transfected into 90% confluent BSR‐T7/5 cells in 6‐well plates using Lipofectamine 3000 per manufacturer's instructions. Viral cytopathic effects appeared by 72 h post‐transfection; supernatants containing progeny virions were harvested for titration and characterization. Virus stocks were amplified in BSR cells and titrated by TCID_50_ assay. All the experiments involving live BTV manipulation in this study were performed in the biosafety level 3 facilities (BSL3) at Harbin Veterinary Research Institute (HVRI) of the Chinese Academy of Agricultural Sciences (CAAS), approved by China's Ministry of Agriculture and Rural Affairs.

### 5.2 Antibodies and Plasmids

All custom antibodies were generated in‐house, including mouse monoclonal antibodies against BTV NS1 and VP7, mouse polyclonal serum against BTV NS3, rabbit polyclonal serum against BTV NS3, mouse polyclonal serum against EHDV VP6, rabbit polyclonal serum against EHDV NS1, and mouse polyclonal serum against EHDV NS3. Commercially sourced antibodies included mouse anti‐Flag, rabbit anti‐HA, mouse anti‐His, rabbit anti‐ZO‐1, rabbit anti‐Myc, and mouse anti‐actin monoclonal antibodies (Proteintech), as well as rat anti‐CD31 monoclonal antibody (Abcam). For detection, the following highly cross‐adsorbed secondary antibodies were used: Goat anti‐mouse/rabbit IgG (H+L) conjugated to Alexa Fluor 488 (A‐11029/A‐11034, Invitrogen) or Alexa Fluor 568 (A‐11031/A‐11011, Invitrogen), donkey anti‐goat IgG (H+L) conjugated to Alexa Fluor Plus 488 (A32814, Invitrogen), and HRP‐conjugated anti‐mouse/rabbit IgG (Invitrogen). For infrared imaging, IRDye 680RD goat anti‐mouse IgG (926‐68070), IRDye 680RD goat anti‐rabbit IgG (926‐68071), IRDye 800CW goat anti‐mouse IgG (926‐32210), and IRDye 800CW goat anti‐rabbit IgG (926‐32211) were obtained from LI‐COR.

The cDNAs encoding C‐terminal 2 × HA‐3 × Flag‐tagged orbivirus NS3 proteins, along with EHDV NS3 5‐alanine scanning mutants, EHDV‐7 NS3 mut1/mut2, BTV‐20 NS3 mut1/mut2, EHDV‐7 NS3_N149Q_, EHDV‐7 NS3_K179A_, and BTV NS3_K183A_, were synthesized by GenScript (Piscataway, NJ) and cloned into a high‐copy pCAGGS vector containing a (GGGGS)_3_ linker between the fusion tag and target gene. Additionally, cDNAs encoding EHDV‐7 NS3 fused with HiBit at the N‐terminus, C‐terminus, or extracellular domain, Flag‐tagged Arf6 Q67L, mWasabi‐tagged PH‐PLCδ, and Myc‐tagged Sec13 were constructed in‐house. Organelle markers (pmCherry‐Calnexin‐ER [#55005] and pmCherry‐TGN‐Golgi [#55145]) were obtained from Addgene. All plasmids were verified by Sanger sequencing (Seven Innovation Biotechnology, China). For transient transfection, HEK‐293T cells (0.6 × 10^6^ cells/well in 12‐well plates) were reverse‐transfected with 1.5 µg plasmid DNA using Lipofectamine 3000 (Thermo Fisher Scientific) per the manufacturer's instructions. Cells were harvested 20 h post‐transfection for analysis.

### 5.3 Viral Infection (In Vitro)

For BTV‐20 and EHDV‐7 infections, BHK‐21/MDOK cells were seeded in 12‐well plates at 0.6 × 10^6^ cells/well. After 12 h incubation, cells were infected with EHDV‐7, BTV‐20 wild‐type (WT), or BTV NS3 _K183A_ at an MOI of 5 in 500 µL serum‐free DMEM for 1 h at 37°C. Post‐adsorption, the inoculum was replaced with 1 mL DMEM containing 2% FBS. For NS3 secretion analysis, supernatants and cells were harvested at 0, 12, 15, 18, and 20 h post‐infection (hpi) for western blotting. Viral replication kinetics were assessed by collecting samples at 4, 8, 12, 16, 20, 24, 28, and 32 hpi for qRT‐PCR.

### 5.4 Viral Infection (In Vivo)

For AG129 mice, six‐to‐eight‐week‐old animals were infected subcutaneously with 1 × 10^3^ TCID_50_ of BTV WT or BTV NS3_K183A_ in a volume of 100 µL. Mock‐infected mice received an equal volume of PBS. Following infection, mice were monitored daily for 14 days to assess body weight changes, clinical signs, and survival. For viremia quantification, blood samples were collected at indicated time points, and viral RNA was extracted and analyzed by qRT‐PCR. At 5 days post‐infection, mice were euthanized, and tissues were collected for viral load quantification and histopathological analysis. For therapeutic evaluation, AG129 mice infected with BTV WT received daily intravenous injections of neomycin (7.5 mg/kg/day) or PBS (negative control); survival was monitored daily for 14 days.

For BALB/c suckling mice, three‐ to five‐day‐old animals were inoculated intracerebrally with 1 × 10^3^ TCID_50_ of BTV WT or BTV NS3_K183A_ in a volume of 20 µL. Mock‐infected mice received an equal volume of PBS. Following inoculation, mice were monitored daily for 14 days to record survival.

For Merino sheep, three‐month‐old animals were infected intravenously with 1 × 10^6^ TCID_50_ of BTV WT or BTV NS3_K183A_ in a volume of 2 mL. Mock‐infected sheep received an equal volume of PBS. Following infection, animals were monitored daily for 14 days to record rectal temperatures and clinical signs. Blood samples were collected at the indicated time points for viremia quantification by qRT‐PCR.

### 5.5 Detection of Secreted NS3

To detect secreted NS3, culture supernatants were clarified by centrifugation at 2000 × g for 10 min at 4°C to remove cellular debris. Clarified supernatants were concentrated ∼10‐fold using 10 kDa centrifugal filters (Millipore). Concentrated samples were mixed with 5 × SDS sample buffer to achieve 1 × final concentration, followed by boiling at 100°C for 15 min. For cell lysis, cells were incubated with ice‐cold lysis buffer (Beyotime, P0013) for 10 min on ice, then centrifuged at 12 000 × g for 10 min at 4°C. The resulting supernatants were combined with 5 × SDS sample buffer (Beyotime, P0015L) to 1 × final concentration and boiled as above. Proteins from both concentrated supernatants and cell lysates were analyzed by western blotting.

### 5.6 Assessment of Plasma Membrane Integrity by Trypan Blue Exclusion

Plasma membrane integrity was assessed by trypan blue exclusion. Cell suspensions were mixed 1:1 (v/v) with 0.4% trypan blue (Invitrogen) and incubated for 3 min at room temperature (RT). Subsequently, 10 µL of the mixture was loaded onto a Countess II FL chamber slide (Invitrogen). Viable cell counts were determined using the Countess II FL Automated Cell Counter (Invitrogen), with membrane‐compromised cells staining blue.

### 5.7 qRT‐PCR Assay for Viral RNA

Viral RNA extraction was performed using the SimplyP Total RNA Extraction Kit (Bioflux, China), followed by quantification via qRT‐PCR with the HiScript II U+ One Step qRT‐PCR Probe Kit (Vazyme, China) on an ABI QuantStudio 5 system (Applied Biosystems, USA). All primer and probe sequences are provided in Table S1.

### 5.8 Rate‐Zonal Centrifugation in Sucrose Gradients

Clarified supernatants from EHDV‐infected BHK‐21 cells or EHDV NS3‐transfected HEK‐293T cells were prepared by centrifugation at 2000 × g for 10 min and concentrated 10‐fold using 10 kDa centrifugal filters (Amicon Ultra, Millipore). Concentrated samples were layered onto 10%–60% sucrose gradients prepared in HBSS (Gibco, 14025–092) and equilibrated overnight at 4°C to form continuous gradients. Ultracentrifugation was performed using a Beckman Optima XPN‐100 with 55Ti rotor at 167 000 × g (42 000 rpm) for 2 h at 4°C. Gradient fractions from EHDV‐infected samples were analyzed by EHDV‐specific qRT‐PCR, virus titration, and western blotting for NS3, VP6, and NS1 proteins, while fractions from NS3‐transfected cells were assessed by western blotting using anti‐NS3 antibodies. Sucrose densities were determined by refractometry.

### 5.9 Electron Microscopy (EM)

EHDV‐infected BHK‐21 cells were harvested by centrifugation at 2000 × g for 10 min and resuspended in 1.5 mL microcentrifuge tubes. Following supernatant removal, cells were sequentially fixed with 4% paraformaldehyde (PFA) and then with a mixed fixative solution containing 2% PFA, 0.1% glutaraldehyde, and 0.2% HEPES. Ultrathin sections were mounted on nickel grids, blocked with 0.1% BSA for 30 min at room temperature (RT), and subjected to immunogold labeling by overnight incubation at 4°C with EHDV NS3 mouse polyclonal antibody (1:200 in PBS) followed by 5 nm gold‐conjugated anti‐mouse secondary antibody. Imaging was performed using an H‐7650 transmission electron microscope (Hitachi, Tokyo, Japan) at 80 kV.

For negative staining, fraction seven samples were applied to glow‐discharged 400‐mesh Formvar/carbon‐coated copper grids, air‐dried, and stained with 2% phosphotungstic acid. Grids were inverted onto droplets of 1% glutaraldehyde in 0.15 m sodium phosphate buffer (pH *7.4*) for 1 min. After blocking with 0.1% BSA (30 min, RT), immunogold labeling was performed using identical antibody conditions and microscope parameters as described above.

### 5.10 Virus Titration by TCID_50_ Assay

Viral infectivity was quantified by tissue culture infectious dose 50 (TCID_50_/mL) assay. Serial ten‐fold dilutions (10^−^^1^ to 10^−^^9^) of viral suspensions were prepared in DMEM supplemented with 2% fetal bovine serum (FBS) and inoculated onto confluent BSR cell monolayers in 96‐well plates. Following a 7‐day incubation at 37°C with 5% CO_2_, titers were calculated using the Spearman‐Kärber method [61].

### 5.11 Confocal Microscopy Analysis

To investigate the subcellular localization of EHDV NS3 wild‐type (WT) and K179A mutant, BHK‐21 cells were co‐transfected with Flag‐tagged NS3 constructs alongside organelle markers: mCherry‐Calnexin (ER), mCherry‐TGN38 (Golgi), GFP‐PH‐PLCδ (PIP2), and Myc‐tagged Sec13. At 20 h post‐transfection, cells were fixed with 4% paraformaldehyde (15 min, room temperature), permeabilized with 0.1% Triton X‐100 in PBS (15 min), and blocked with 10% goat serum (1 h, 37°C). Sequential immunostaining was performed using mouse anti‐Flag (1:200) and rabbit anti‐Myc (1:200) primary antibodies (overnight, 4°C), followed by species‐matched Alexa Fluor‐conjugated secondary antibodies (1:1000, 1 h, room temperature) and DAPI nuclear counterstain (1:1000). Images were acquired using a Zeiss LSM 800 confocal microscope with 63 × oil immersion objective.

For endothelial barrier function assays, confluent bovine pulmonary artery endothelial cell (CPAE, ATCC CCL‐209) monolayers were treated for 24 h with either 20 µg/mL MBP (negative control) or HEK‐293F‐derived BTV NS3 protein. Tight junction integrity was assessed by immunofluorescence staining with rabbit polyclonal anti‐ZO‐1 antibody using the aforementioned protocol. Sialic acid distribution was evaluated by incubation with Alexa Fluor 488‐conjugated wheat germ agglutinin (WGA, 30 min, 37°C), followed by Hoechst 33342 nuclear staining (10 min, room temperature). All imaging was performed under standardized acquisition parameters on the Zeiss LSM 800 system.

### 5.12 Co‐Immunoprecipitation Assay (Co‐IP)

HEK‐293T cells were transfected with plasmids encoding Flag‐tagged NS3 and Myc‐tagged Sec13. At 24 hpt, cells were washed three times with ice‐cold PBS and lysed using NP‐40 lysis buffer (Beyotime, P0013) on ice. The lysates were incubated overnight at 4°C with anti‐Flag antibody‐conjugated beads (Proteintech 66008‐4‐Ig). After three washes with lysis buffer, immunoprecipitated complexes were eluted and analyzed by western blotting.

### 5.13 NS3 Expression and Purification

The NS3 genes from both BTV and EHDV were cloned into the pTT5 expression vector (National Research Council Canada) with an N‐terminal maltose‐binding protein (MBP) tag. Recombinant protein expression was carried out in HEK‐293F cells transfected with the ExpiFectamine 293 Transfection Kit (Thermo Fisher Scientific) according to the manufacturer's protocol. At 72 hpt, cell cultures were harvested by centrifugation at 300 × g for 10 min, and supernatants containing secreted NS3 proteins were filter‐sterilized through 0.2 µm PVDF membranes (Merck Millipore). The filtered supernatants were then concentrated ∼10‐fold using 10 kDa ultrafiltration membranes (Merck Millipore).

Immunoaffinity purification was conducted as previous reported with critical modifications [62]. Briefly, 5 mL of AminoLink Plus coupling resin (Thermo Fisher Scientific) pre‐conjugated with NS3‐specific mouse polyclonal antibodies was incubated with the concentrated supernatant under constant rotation overnight at 4°C. The resin‐protein complexes were then packed into an Econo‐Pac chromatography column (Bio‐Rad) and washed extensively with 10 column volumes of sterile PBS (pH 7.4). Bound proteins were eluted with 3 column volumes of 0.1 m glycine‐HCl (pH *2.7*), followed by immediate neutralization with 1 M Tris‐HCl (pH *9.0*). The eluates were dialyzed against PBS (pH *7.4*) overnight at 4°C, concentrated using 10 kDa ultrafiltration membrane (Merck Millipore), and verified to be >95% pure by SDS‐PAGE analysis. Purified proteins were aliquoted and stored at −80°C for downstream applications.

### 5.14 LDH Release Assay

Cells were seeded in 96‐well plates at 1 × 10^5^ cells/well and transfected with 0.2 µg/well of EHDV NS3 WT or mutant plasmids. LDH release assays were performed 20 hpt using the LDH Cytotoxicity Detection Kit (Beyotime, C0016), with controls including cell‐free medium (background control), untransfected cells (sample background), and lysed untreated cells (maximum activity control). For maximum activity controls at 19 hpt, medium was aspirated, wells were washed with PBS and replaced with fresh medium containing 1% FBS. LDH Release Reagent (10% v/v) was added followed by 1 h incubation at 37°C. After centrifugation (400 × g, 5 min), 120 µL supernatant/well was transferred to a new plate, mixed with 60 µL LDH detection solution, and incubated 30 min at 25°C in the dark with agitation. Absorbance at 490 nm was measured using a microplate reader, and membrane permeability (%) was calculated as [(OD treated – OD sample background)/ (OD maximum activity – OD sample background)] × 100%.

### 5.15 Cell Counting Kit‐8 (CCK‐8) Assay

Cells were seeded in 96‐well plates at 1 × 10^5^ cells/well and transfected with 0.2 µg/well of EHDV NS3 WT or K179A mutant expression plasmids. Cell viability was assessed 20 hpt using the Cell Counting Kit‐8 (CCK‐8; APExBIO, K1018, USA), with 10 µL of CCK‐8 solution carefully added to each well while avoiding air bubble formation. Plates were incubated at 37°C/5% CO_2_ for 2 h, and absorbance at 450 nm was measured using a SpectraMax microplate reader (Molecular Devices). Relative cytotoxicity (%) was calculated as [1 – (OD from tested cells)/OD from control cells] × 100%.

### 5.16 Evans Blue Dermal Miles Assay

The effect of BTV and EHDV NS3 proteins on vascular permeability in vivo was evaluated using a modified Miles assay [63]. BALB/c mice were shaved three days before the experiment. On the day of the assay, mice were injected retro‐orbitally with Evans Blue dye (EBD; 0.5% in PBS, 150 µL). After 1.5 h, mice were anesthetized, and MBP (20 µg in 50 µL), VEGF (200 ng in 50 µL), and purified BTV/EHDV NS3 (10 µg or 20 µg in 50 µL) were injected intradermally at distinct sites on the shaved dorsal skin. At 0.5 h post‐injection, mice were euthanized, and the dorsal skin containing the injection sites was excised. The areas of Evans Blue leakage were carefully dissected and incubated in formamide at 56°C for 24 h. The absorbance of the extravasated dye was measured at 630 nm using a spectrophotometer.

### 5.17 Evans Blue Systemic Mile's Assay

AG129 mice were infected with either wild‐type BTV or the S10K183A mutant at a dose of 10^3^ TCID_50_ per mouse. Five days post‐infection, mice were intravenously injected with 150 µL of 0.5% Evans Blue dye (EBD in PBS) and allowed to circulate for 2 h. After euthanasia, tissues were collected via cardiac puncture, dried, and placed in pre‐weighed tubes. One milliliter of formamide was added to each tube, and samples were incubated at 56°C for 24 h. The absorbance of the extravasated dye was measured at 630 nm using a spectrophotometer.

### 5.18 Trans‐Endothelial Electrical Resistance (TEER)

The effect of recombinant orbivirus NS3 on endothelial permeability was assessed by measuring by TEER in bovine endothelial cells grown in a 24‐well Transwell system (0.4 µm pores, 6.5 mm inserts; Corning Inc.). TEER values (in Ohms, Ω) were measured at two‐hour intervals following the addition of test proteins using an Epithelial Volt Ohm Meter (EVOM) with “chopstick” electrodes (World Precision Instruments). Untreated endothelial cells served as negative controls, while inserts containing medium alone were used for blank resistance measurements. Relative TEER was calculated using the formula: [(Ω_{experimental} – Ω_{medium}) / (Ω_{non‐treated} – Ω_{medium})]. After 24 h, 50% of the media in both the upper and lower chambers was replaced with fresh medium. For experiments involving monoclonal antibodies (mAbs) and inhibitors, mAbs or inhibitors were mixed with test proteins and added to the upper chamber.

### 5.19 Histopathological Analysis

Organ tissues from mice infected with BTV WT or BTV NS3_K183A_ were fixed in 10% neutral buffered formalin for 3 days, embedded in paraffin, and sectioned into 5‐µm slices. The sections were stained with hematoxylin and eosin (H&E), photographed using an Olympus CX31 microscope (Olympus Corporation), and analyzed.

### 5.20 Liposome Sedimentation Assays

Multilamellar vesicles containing defined ratios of phosphatidylcholine (PC) and phosphatidylinositol 4,5‐bisphosphate (PIP2) were prepared as described previously [44]. Purified BTV NS3 and MBP proteins were centrifuged at 20 000 × g, 4 °C for 30 min, after which the supernatant was transferred to a fresh tube for protein quantification. Aliquots of 50 µL BTV NS3 (100 µm), 50 µL MBP (100 µm), and 50 µL liposomes were combined and mixed gently. The mixture was incubated at room temperature (RT) for 30 min, followed by centrifugation at 20 000 × g, 22 °C for 30 min. The supernatant was transferred to a new tube containing 25 µL of 5× loading buffer, while the pellet was resuspended in 12 µL of 1× loading buffer. All samples were denatured at 100 °C for 15 min prior to analysis by SDS‐PAGE and western blotting.

### 5.21 De‐Glycosylation Assays

N‐linked glycosylation from orbivirus NS3 protein was removed using PNGase F (P0704, NEB, USA). Cell lysates or supernatants (18 µL) were mixed with 2 µL of 10 × Glycoprotein Denaturing Buffer and denatured at 100°C for 10 min. After cooling, the reaction was supplemented with 4 µL of 10× GlycoBuffer 2, 4 µL of 10% NP‐40, 2 µL PNGase F, and 10 µL distilled water, followed by incubation at 37 °C for 1 h. The sample was then mixed with 5 × SDS reducing buffer (final concentration: 1 ×) and boiled at 100 °C for 15 min. Deglycosylated proteins were analyzed by western blotting.

### 5.22 Characterization of NS3 Membrane Encapsulation Status

To determine whether secreted NS3 was fully enveloped by lipid membranes, proteinase K (Beyotime, ST535) and NP‐40 were used in combination. Proteinase K, as a nonspecific protease, digests exposed proteins into small peptides but cannot access proteins completely enclosed within intact lipid membranes. When NP‐40 was added to disrupt membranes, proteinase K gained access to and degraded the proteins. Protein samples were treated with 1% NP‐40 at 37°C for 15 min or left untreated, followed by incubation with proteinase K (100 µg/mL) at 37°C for 15 min to degrade accessible proteins. The reaction products were analyzed by western blotting.

### 5.23 SDS‐PAGE and Western Blotting Assay

The collected protein samples were separated using 10% sodium dodecyl sulfate‐polyacrylamide gel electrophoresis (SDS‐PAGE). For SDS‐PAGE analysis, the gel was stained with QuickBlue Rapid Staining Solution (Biodragon, BF06152) for 10 min under gentle agitation to visualize protein bands. For western blotting analysis, separated proteins were transferred onto nitrocellulose membranes (Thermo Fisher Scientific, STM2007) using an eBlot L1 Transfer System (GenScript, China). The membranes were blocked with 5% skimmed milk in PBS containing 0.1% Tween‐20 (PBST) for 1 h at room temperature, followed by overnight incubation at 4°C with primary antibodies diluted in PBST containing 1% skimmed milk. Primary antibodies included: mouse anti‐FLAG mAb (for transfected NS3/Arf6 Q67L), BTV/EHDV NS3‐specific mouse polyclonal serum (for viral NS3), and mouse anti‐MBP mAb (for recombinant proteins). After four 5‐min PBST washes, membranes were incubated with species‐matched IRDye secondary antibodies in 1% milk‐PBST for 1 h at RT, followed by imaging on an Odyssey CLX system (LI‐COR) with simultaneous 700/800 nm detection.

### 5.24 Nano‐Glo HiBiT Extracellular Detection Assay

HEK‐293T cells were seeded at 2 × 10^4^ cells/well in 96‐well plates and cultured overnight. Transfection was performed with 0.1 µg of the following plasmids: pCAGGS‐NS3/3A, pCAGGS‐HiBiT‐NS3/3A, pCAGGS‐NS3/3A‐HiBiT (ED), or pCAGGS‐NS3/3A‐HiBiT. After 20 h, 100 µL supernatant was collected, centrifuged at 2000 × g for 10 min, and transferred to fresh 1.5‐mL tubes. Cells were harvested with 100 µL PBS, transferred to 1.5‐mL tubes, and lysed with 0.05% Triton X‐100 at RT for 10 min. For luminescence assays, 50 µL supernatant or lysate was transferred to white opaque 96‐well plates, mixed with 50 µL Nano‐Glo HiBiT Extracellular Reagent (containing buffer, substrate, and LgBiT protein), and incubated for 10 min. Luminescence was measured using a microplate reader, and extracellular NS3 proportion was calculated as: Proportion of extracellular NS3 = (Extracellular Test‐Extracellular Background) / (Lytic Test – Lytic Background).

### 5.25 BTV NS3 Detection by Capture ELISA

For detection of BTV NS3 in infected mouse serum, ELISA plates were coated with mouse anti‐BTV NS3 polyclonal antibodies (1:200 dilution in coating buffer) and incubated at 37°C for 1 h. After three 5‐min PBST washes, plates were dried and blocked with 200 µL/well of 5% non‐fat milk in PBS at 37°C for 1 h, followed by three additional PBST washes. Protein standards and test samples (100 µL/well) were then added and incubated at 37°C for 1 h. Following washing, rabbit anti‐BTV NS3 polyclonal antibodies (1:200 in PBS) were added and incubated for 1 h at 37°C. After further washing, HRP‐conjugated anti‐rabbit IgG (1:5000 dilution) was added for 1 h incubation. Finally, 50 µL/well of HRP substrate was added for 10 min, the reaction was stopped with 50 µL/well stop solution, and absorbance was measured at 450 nm using a microplate reader.

### 5.26 RNA‐Seq Data Generation

AG129 mice were either mock‐infected or infected with BTV WT or BTV NS3_K183A_, then were sacrificed at 5 dpi. Total RNA from spleen and lung tissues was purified, and sequencing libraries were prepared using the Illumina TruSeq protocol and analyzed on an Illumina HiSeq platform. Four biological replicates were included per group, and differentially expressed genes were identified using DESeq2 with default parameters.

### 5.27 Statistical Analysis

All statistical analyses were performed using GraphPad Prism (v10.1.2). Data preprocessing included normalization of measured values to the respective control group for quantitative assays (e.g., Western blot, qPCR, HiBiT assay, TEER, Evans Blue extravasation, and cell viability assays). For RNA‐seq data, log2 transformation was applied prior to differential expression analysis. Outliers were evaluated using the interquartile range (IQR) method. Sample sizes (n) are specified in each figure legend.

All quantitative data are presented as mean ± standard deviation (SD). Comparisons between two groups were analyzed using two‐tailed unpaired Student's *t*‐test. For comparisons involving more than two groups, one‐way or two‐way analysis of variance (ANOVA) was used, followed by Dunnett's post hoc test for multiple comparisons against the control group. All experiments were performed with at least three biological replicates unless otherwise stated.

### 5.28 Image Generation

Schematic illustrations were created with BioRender (https://www.biorender.com/library).

## Author Contributions

X.Y. designed the experiments. J.G., D.Z., and Y.Q. performed the majority of the experiments and analyzed data with the help of R.S., X.L., Y.H., S.Q., C.X., and M.W. J.G. wrote the manuscript, and X.Y. edited the writing. All authors reviewed, critiqued, and provided comments on the manuscript.

## Funding

This work was supported by the National Key Research and Development Program of China (Grant No. 2024YFD1800102), the National Natural Science Foundation of China (Grant No. 32573398), and the National Natural Science Foundation of China Youth Science Fund (Grant No. 32102651).

## Conflicts of Interest

The authors declare no conflicts of interest.

## Supporting information




**Supporting File**: advs75255‐sup‐0001‐SuppMat.docx.

## Data Availability

All data are included in the manuscript or Supporting Information. RNA‐seq data are available in the European Nucleotide Archive under accession number PRJNA1191894).
