# Supplementary material for: Secreted Nonstructural Protein 3 is a Pathogenic Determinant of Orbivirus
Source: Adv Sci (Weinh). 2026 Apr 13;13(42):e75255. doi: 10.1002/advs.75255 (PMC13334943; doi:10.1002/advs.75255)
Supplement: Supplementary file 1 — Supporting File: advs75255‐sup‐0001‐SuppMat.docx. [file ADVS-13-e75255-s001.docx]

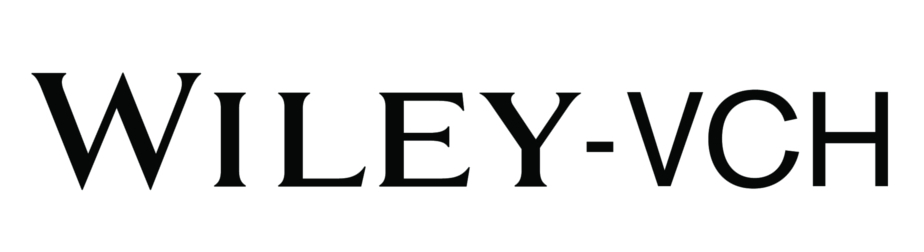


Supporting Information

**Secreted Nonstructural Protein 3 is a Pathogenic Determinant of Orbivirus**

Junyong Guan1^†^, Dong Zhou1^†^, Ran Shao1, Xing Liu1, Jin Peng2, Yingran Huang1, Shuhui Qi1, Cankun Xi1, Menghang Wang1, Bin Sun2*, Yinglin Qi1*, Xin Yin1*

^†^These authors contributed equally to this work

*Corresponding author. Email: [yinxin@caas.cn](mailto:yinxin@caas.cn); [qiyinglin@caas.cn](mailto:qiyinglin@caas.cn); [binsun@hrbmu.edu.cn](mailto:binsun@hrbmu.edu.cn)

Figure S1


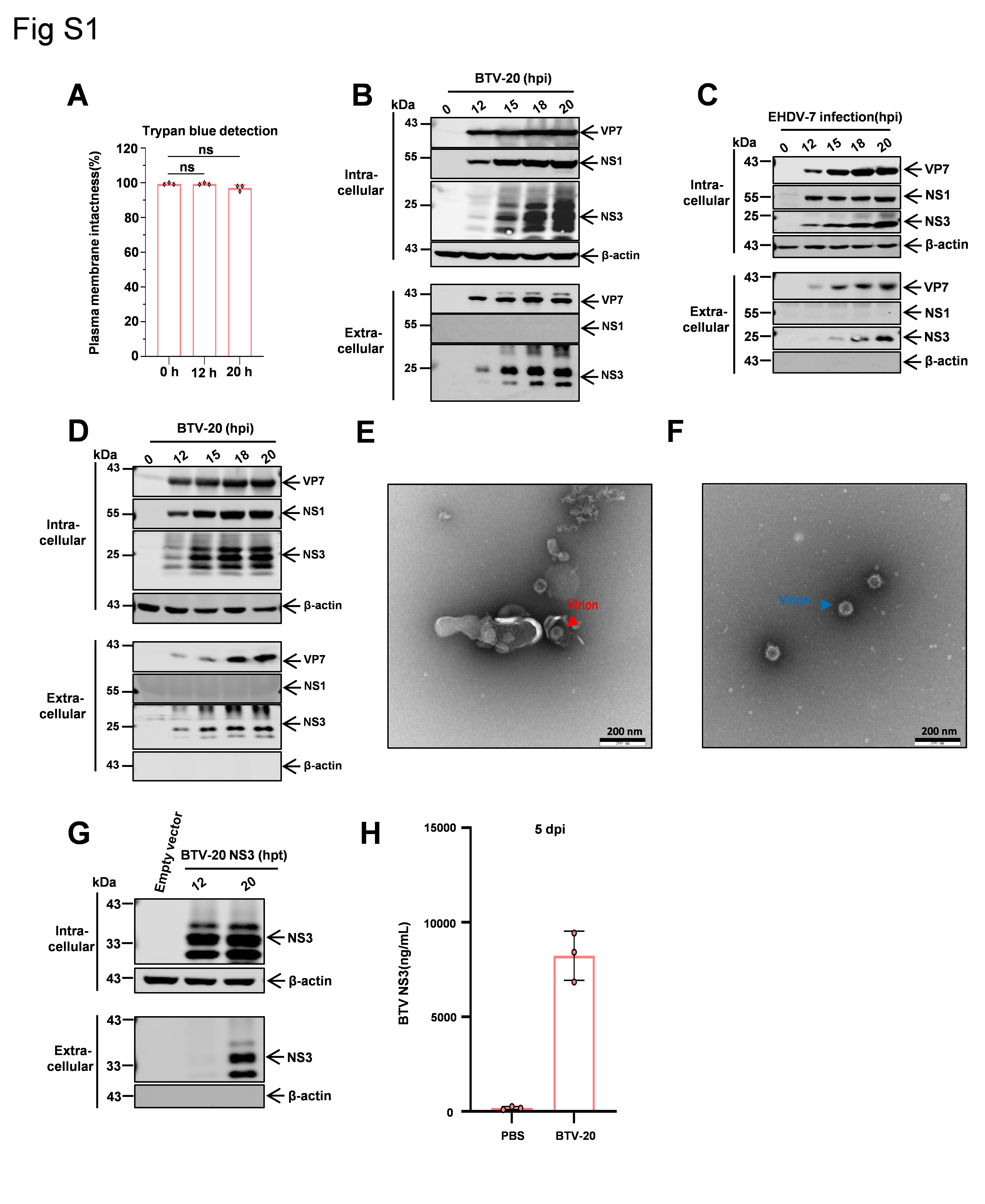


Figure S1. Orbivirus NS3 Possesses a Conserved Capacity for Self-secretion. (A) Plasma membrane integrity of BHK-21 cells infected with EHDV (MOI = 5) was assessed by trypan blue exclusion assay. (B–D) NS3 secretion kinetics post-orbivirus infection: BHK-21 cells (B) or MDOK cells (C–D) were infected with BTV-20 (MOI = 5, B/D) or EHDV-7 (MOI = 5, C). Cells and medium were harvested at 12-20 h post-infection (hpi) for Western blot analysis. (E–F) Electron microscopy of sucrose gradient fractions from (B): (E) Fraction 15 showing membrane-associated virions (red arrows); (F) Fraction 22 with free virions (blue arrows). (G) NS3 secretory capacity validation: HEK-293T cells transfected with BTV-20 NS3 plasmids were harvested at 12/20 h post-transfection (hpt) for Western blot. (H) In vivo NS3 detection: AG129 mice infected subcutaneously with BTV WT (1 × 10^3^ TCID_50_, PBS control) were analyzed for serum NS3 levels by capture ELISA at 5 days post-infection (dpi) (n = 3). The data shown in A are means ± SD (n = 3 biologically independent experiments). Statistical analysis was performed using ordinary one-way ANOVA, followed by Dunnett’s post hoc test for multiple comparisons. *P < 0.05, **P < 0.01, ***P < 0.001, ****P < 0.0001; ns, not significant.

Figure S2


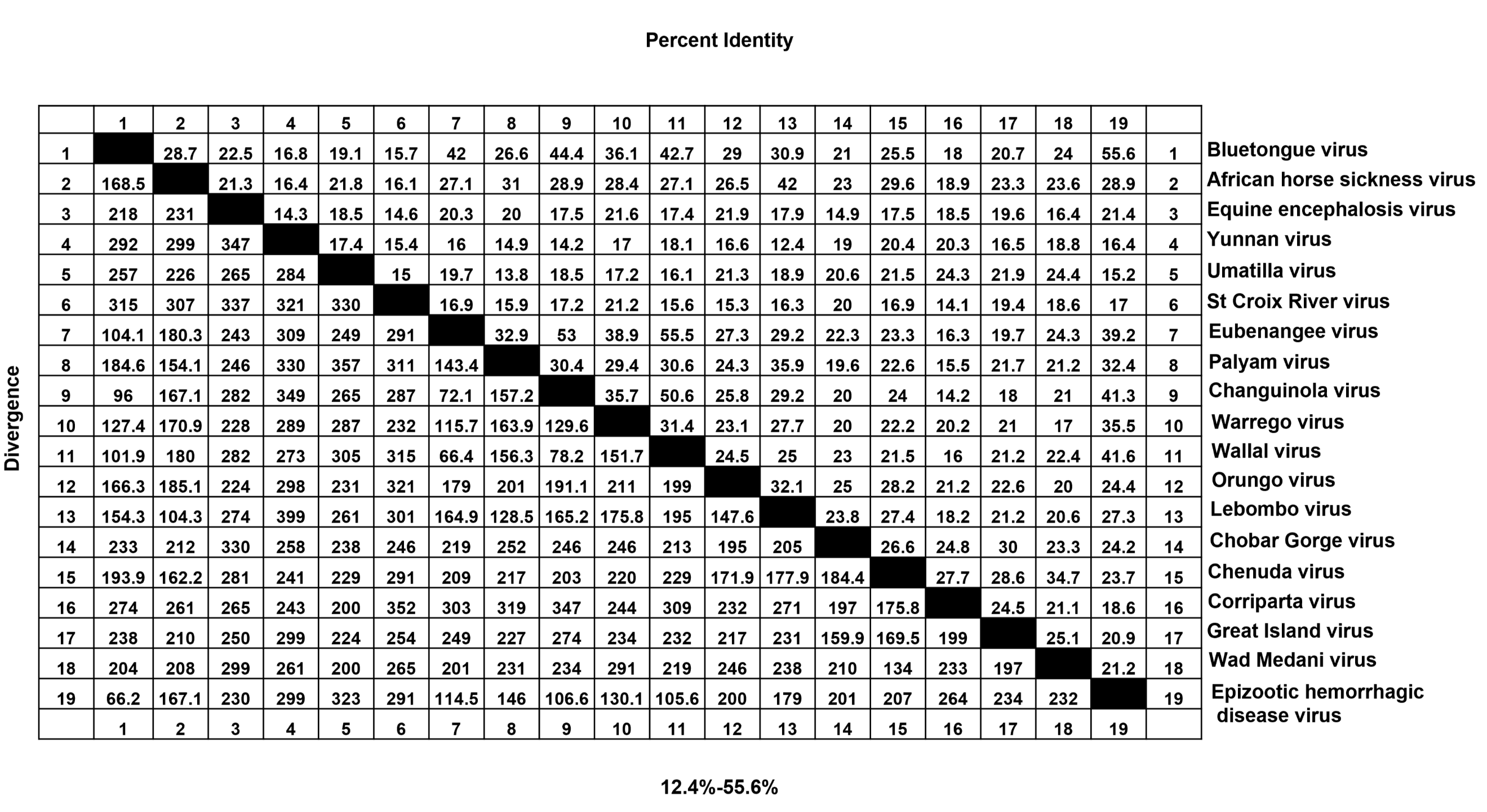


Figure S2. Homology Analysis of Orbivirus NS3. Homology analysis of 19 orbivirus NS3 amino acid sequences was conducted using MEGA 11, indicating the divergence and percent identity among orbivirus NS3 proteins. The GenBank accession numbers of all analyzed orbivirus NS3 are provided in Table S3.

Figure S3


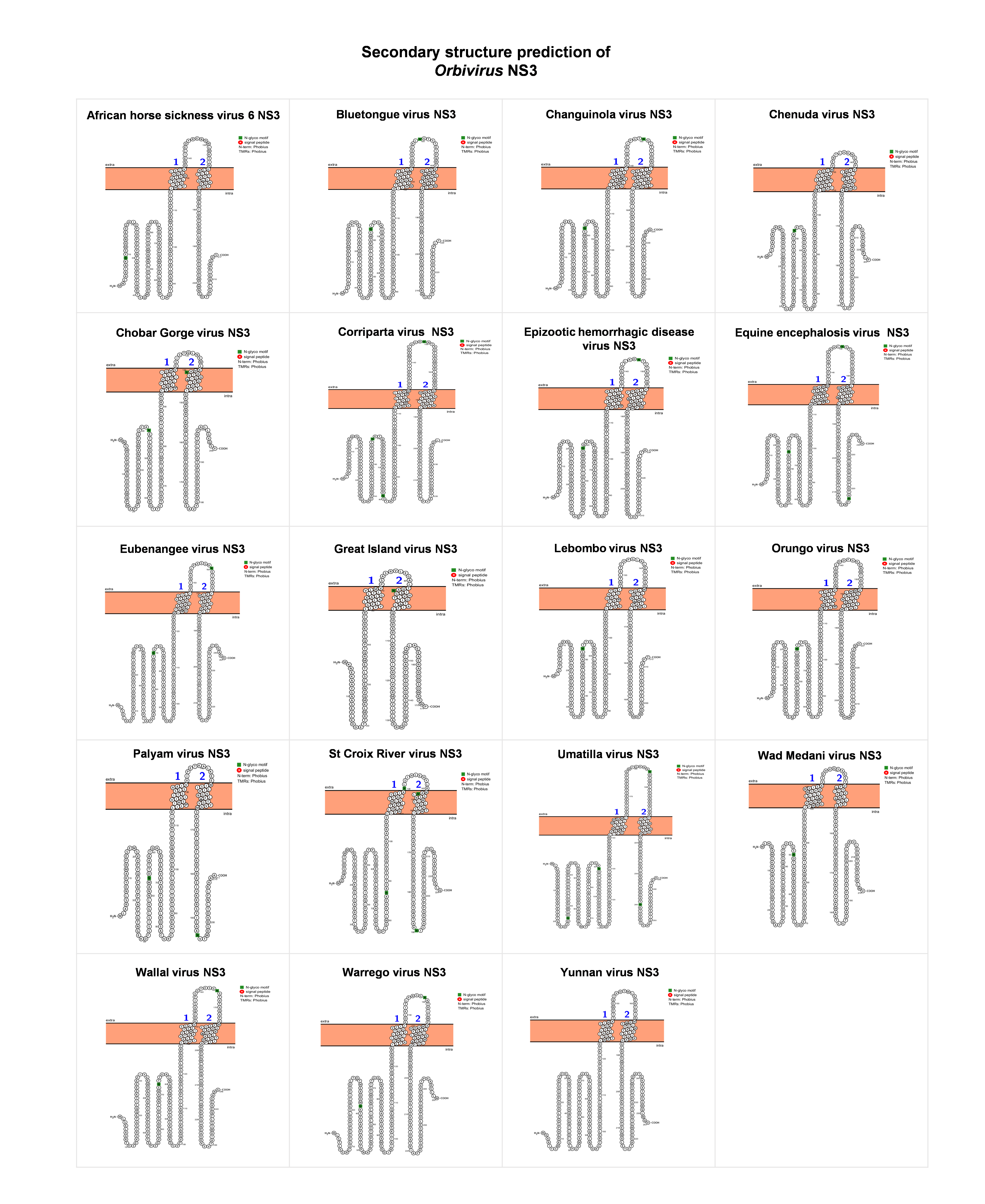


Figure S3. Secondary Structure Prediction of Orbivirus NS3. The secondary features of orbivirus NS3 were predicted by Protter (<https://wlab.ethz.ch/protter/start/>).

Figure S4


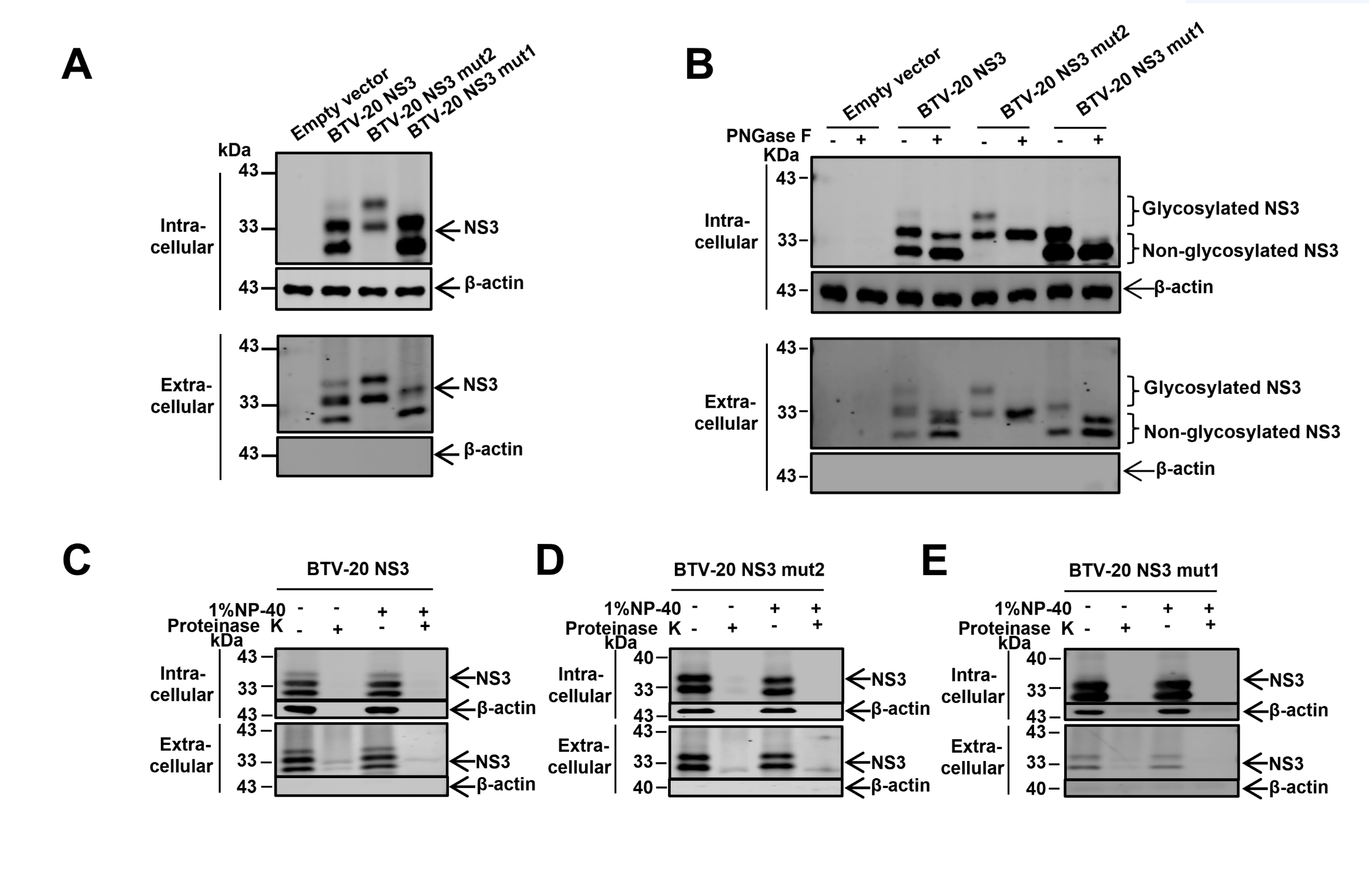


Figure S4. Characterization of Secreted Orbivirus NS3. (A) Characterization of secreted NS3 isoforms: HEK-293T cells were transfected with either empty vector (negative control), BTV-20 NS3 WT, mut1, or mut2, followed by collection of cells and medium at 20 hpt for Western blot analysis. (B) Glycosylation analysis: PNGase F treatment of samples from BTV-20 NS3 WT-, mut1-, and mut2-transfected HEK-293T cells (with PBS as negative control) confirmed NS3 glycosylation status, with glycosylated and non-glycosylated forms indicated. (C–E) Membrane association assessment: Proteinase K and 1% NP-40 treatments were used to evaluate membrane coating of secreted BTV-20 NS3 WT (C), mut2 (D), and mut1 (E), followed by Western blot detection.

Figure S5


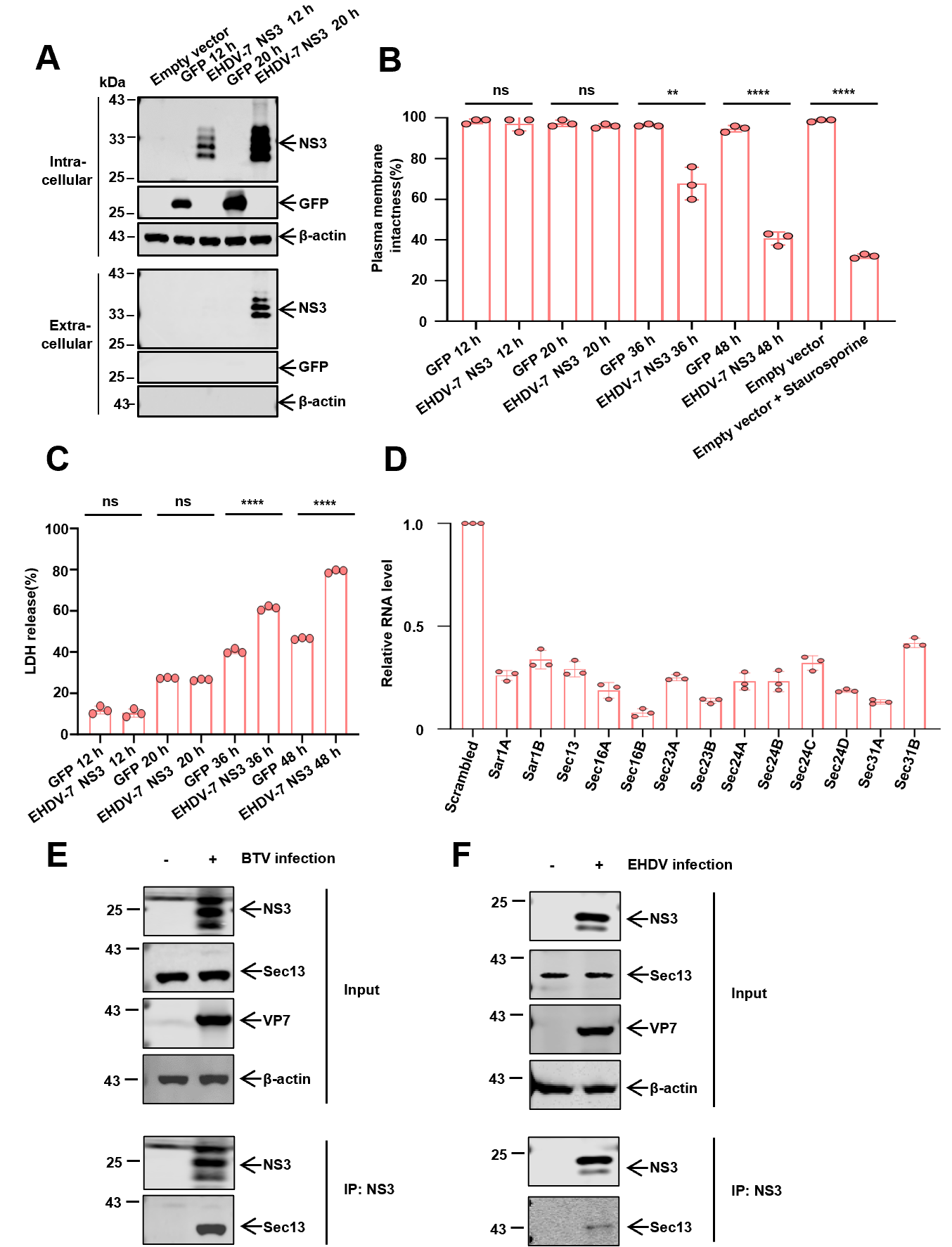


**Figure** **S5.** **Conventional Secretory Pathway is Crucial for NS3 Secretion.** (**A**) Secretory analysis of EHDV-7 NS3: HEK-293T cells transfected with empty vector, EHDV-7 NS3 WT, or eGFP were harvested for Western blot detection of secreted proteins. (**B-C**) Membrane integrity assessment: Trypan blue exclusion assay **(B)** and LDH release assay **(C)** were performed on HEK-293T cells expressing EHDV-7 NS3 or eGFP, with empty vector-transfected cells as negative controls and staurosporine-treated cells as positive controls. (**D**) RNAi screening: HEK-293T cells transfected with NS3 WT or Gaussia luciferase (Gluc) were treated with Sec-targeting siRNAs, followed by qPCR quantification of Sec mRNA levels (primers listed in Supplementary Table S4). (**E–F**) Co-immunoprecipitation (Co-IP) of NS3-Sec13 complexes in BHK-21 cells infected with (**E**) BTV-20 or (**F**) EHDV-7 (MOI = 5, analyzed at 20 hpi). The data shown in **B**–**D** are means ± SD (n = 3 biologically independent experiments). Statistical analysis was performed using two-way ANOVA, followed by Dunnett’s post hoc test for multiple comparisons. *P < 0.05, **P < 0.01, ***P < 0.001, ****P < 0.0001; ns, not significant.

Figure S6


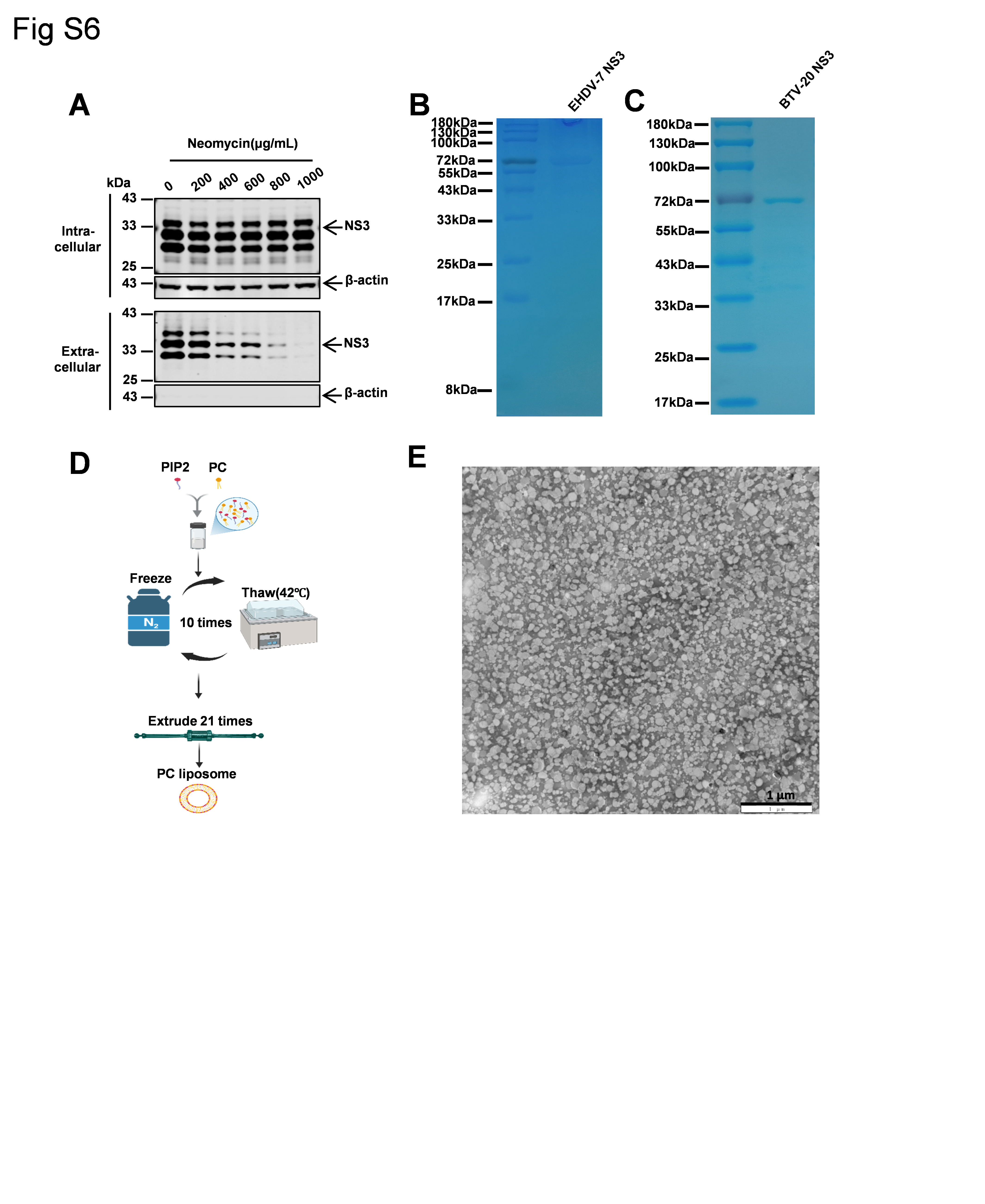


**Figure S6.** **Orbivirus NS3 Purification and Liposome Co-sedimentation Assay.** (**A**) Dose-dependent effects of neomycin (200–1000 μg/mL), a PIP2-binding inhibitor, on BTV NS3 secretion. HEK-293T cells transfected with BTV NS3 plasmids were treated with neomycin at 6 hpt, with cells and medium harvested at 20 hpt. Secreted NS3 levels were analyzed by Western blot. (**B–C**) SDS-PAGE analysis of purified EHDV-7 NS3 (**B**) and BTV-20 NS3 (**C**). (**D**) Schematic of PC liposome preparation. (**E**) Electron micrograph of PC liposomes (scale bar: 1 μm).

Figure S7


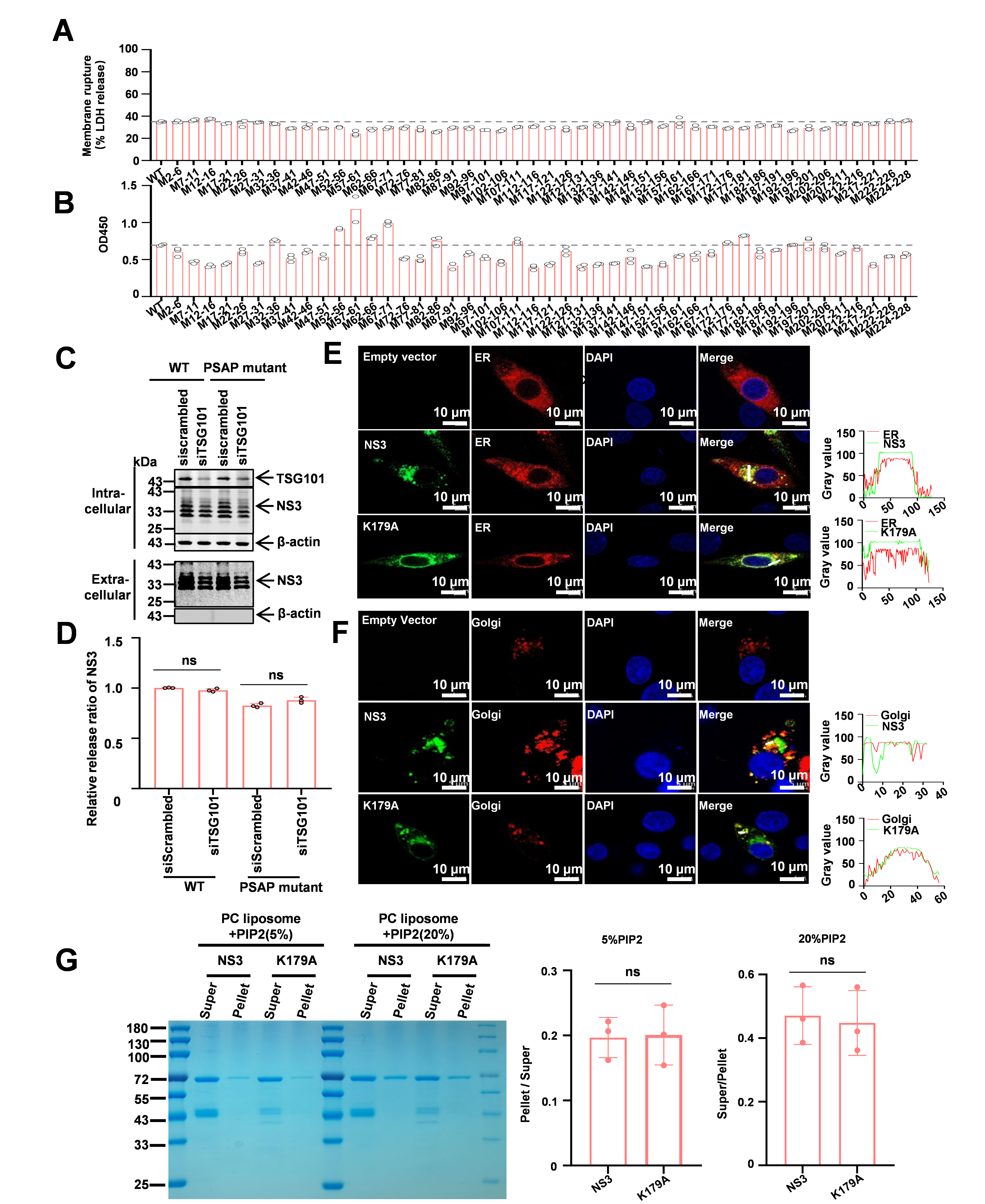


Figure S7. The Critical Role of Highly Conserved K/R Residue Adjacent to the Second Transmembrane Domain in NS3 Secretion. (A–B) Cell viability and membrane integrity of HEK-293T cells expressing EHDV-7 NS3 WT or mutants were assessed by LDH release assay (A) and CCK-8 assay (B). (C) Evaluation of the effect of deficient NS3-mediated viral release on NS3 secretion. The siRNAs targeting TSG101 were transfected into HEK-293T cells, followed by transfection with EHDV-7 NS3 WT or PSAP mutant (M37-41). The secretion levels of NS3 were detected by Western blot. (D) Quantification of (C) using Image J, with NS3 levels normalized to WT+scrambled siRNA controls (set as 1). (E–F) Subcellular localization: Confocal microscopy analysis of EHDV-7 NS3 and K179A mutant colocalization with ER (E) and Golgi (F), with fluorescence signals quantified by ImageJ. (G) Lipid interaction: Liposome co-sedimentation assay evaluating PIP2 binding by EHDV-7 NS3 WT and K179A mutant. The data shown in A–B, D, G are means ± SD (n = 3 biologically independent experiments). Statistical analysis was performed using two-tailed unpaired Student’s t test. *P < 0.05, **P < 0.01, ***P < 0.001, ****P < 0.0001; ns, not significant.

Figure S8


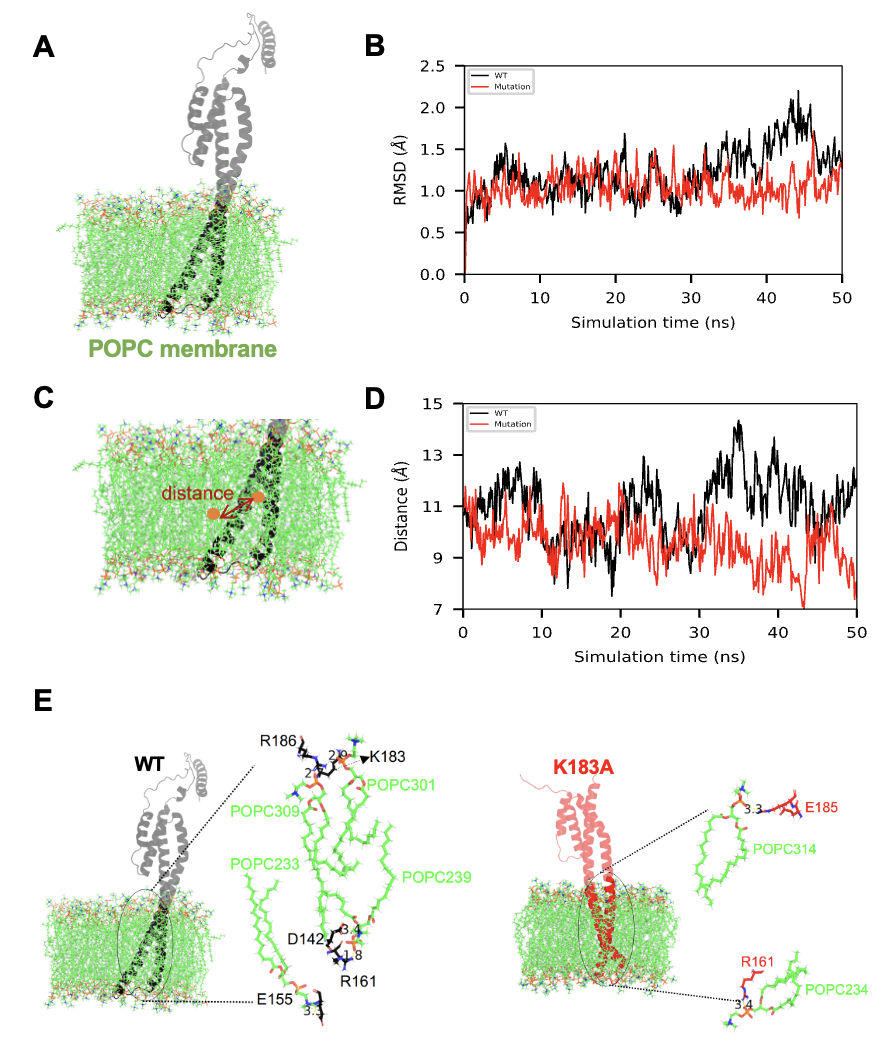


**Figure S8.** **MD simulations of the NS3 protein in a POPC membrane.** (**A**) Schematic of the

simulation setup. (**B**) Time-dependent RMSD values of the transmembrane regions of the

NS3 protein, calculated relative to the starting structure. (**C–D**) The embedding depth of the

protein was gauged by measuring the distance between the center of mass of the protein's

transmembrane regions and the center of mass of the lipids. (**E**) Representative snapshot

structures showing hydrogen bonds between the NS3 protein and the polar head groups of the

lipids.

Figure S9


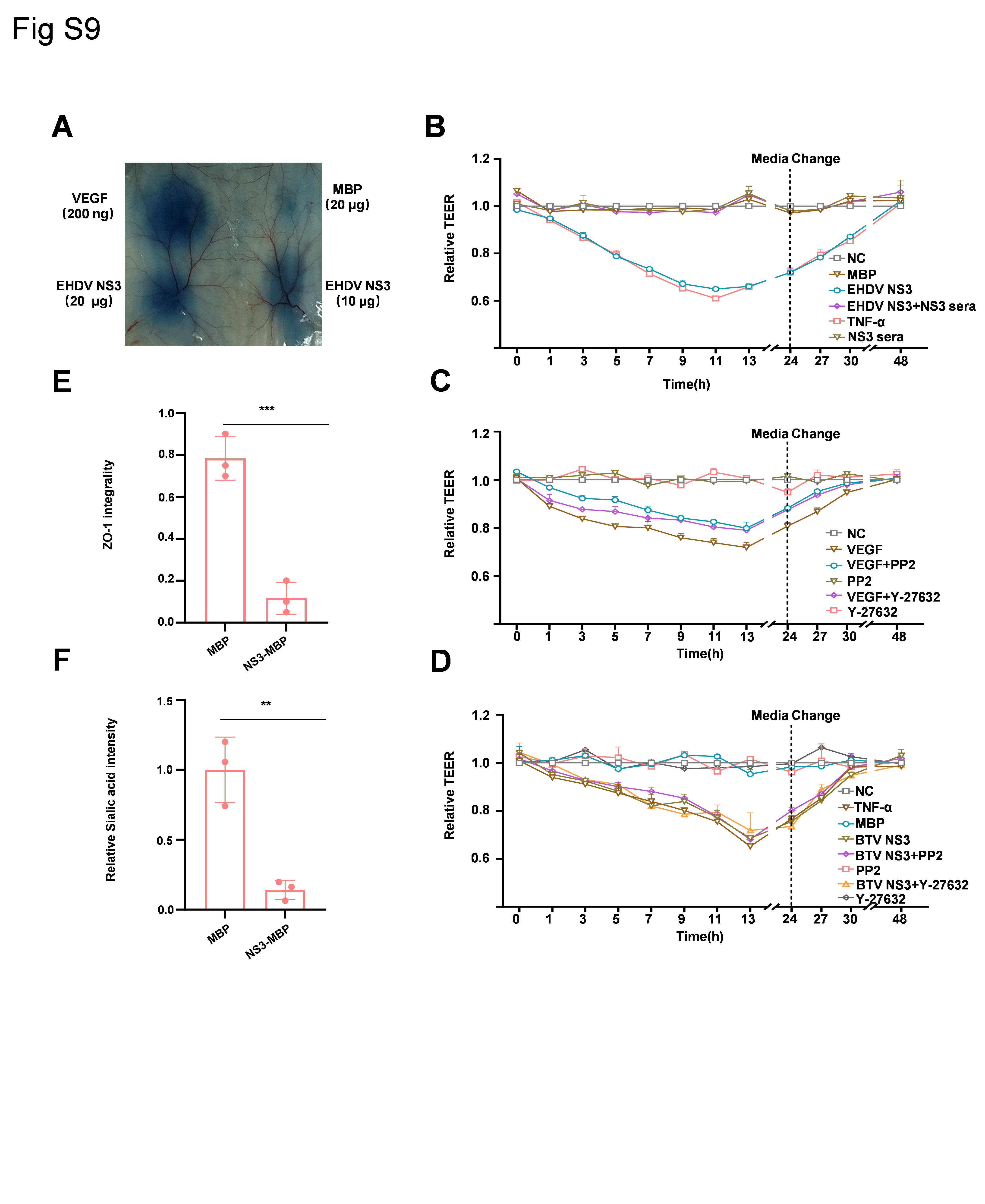


Figure S9. Orbivirus NS3 Triggers Endothelial Permeability and Vascular Leakage. (A) Vascular permeability induction by EHDV-7 NS3 proteins was assessed using the Evans Blue dermal Miles assay. (B) Endothelial barrier disruption was evaluated by transendothelial electrical resistance (TEER) measurements in bovine endothelial cells treated with EHDV-7 NS3. (C–D) TEER assays evaluating endothelial barrier integrity in bovine endothelial cells treated with VEGF (C) or BTV-20 NS3 (D), either alone or in combination with the Src kinase inhibitor PP2 or the ROCK inhibitor Y-27632. (E–F) Quantitative analysis of endothelial junction integrity: (E) ZO-1 organization and (F) sialic acid expression levels from corresponding figures. The data shown in B–F are means ± SD (n = 3 biologically independent experiments). Statistical analysis was performed using two-tailed unpaired Student’s t test. *P < 0.05, **P < 0.01, ***P < 0.001, ****P < 0.0001; ns, not significant.


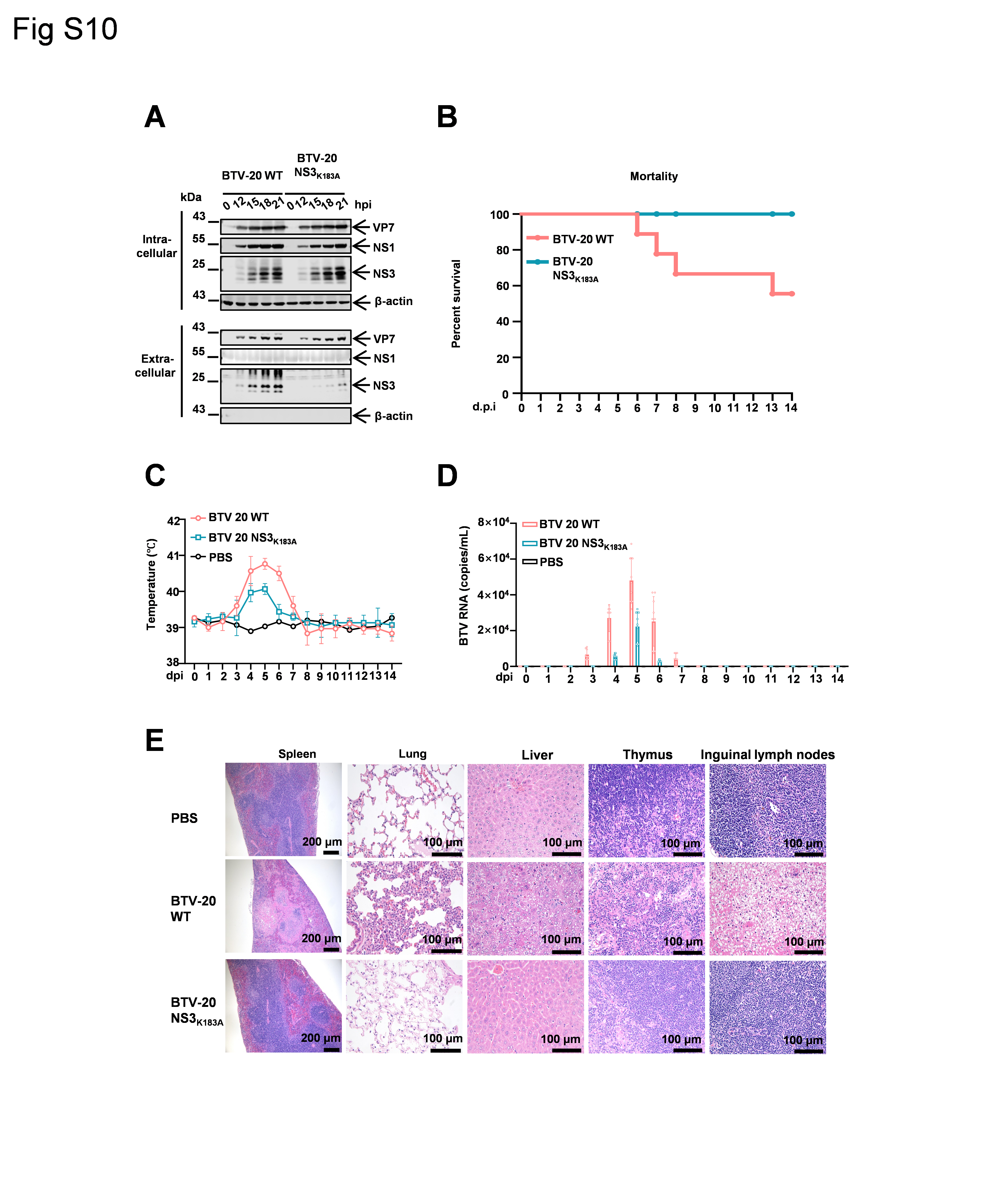
**Figure S10**

Figure S10. Pathogenicity analysis of BTV NS3K183A mutant. (A) Validation of secretory deficiency in BTV NS3_K183A_ mutant: MDOK cells infected with BTV WT or BTV NS3_K183A_ (MOI = 5) were harvested at 12–20 hpi, with NS3 secretion analyzed by Western blot. (B) Survival analysis of BALB/c suckling mice following intracerebral inoculation with BTV WT versus BTV NS3_K183A_ over 14 days post-infection (n = 9).  (C–D) Pathogenicity assessment in Merino sheep, a natural host of BTV. Sheep (n = 3 per group) were infected with BTV-20 WT or BTV-20 NS3K183A and monitored for 14 days. (C) Rectal temperatures were recorded daily. (D) Viremia levels were quantified by qRT-PCR at indicated time points. (E) Pathological analysis: AG129 mice infected subcutaneously with BTV WT or BTV NS3_K183A_ were evaluated by H&E staining of spleen, lungs, liver, thymus, and inguinal lymph nodes at 5 dpi.

Figure S11


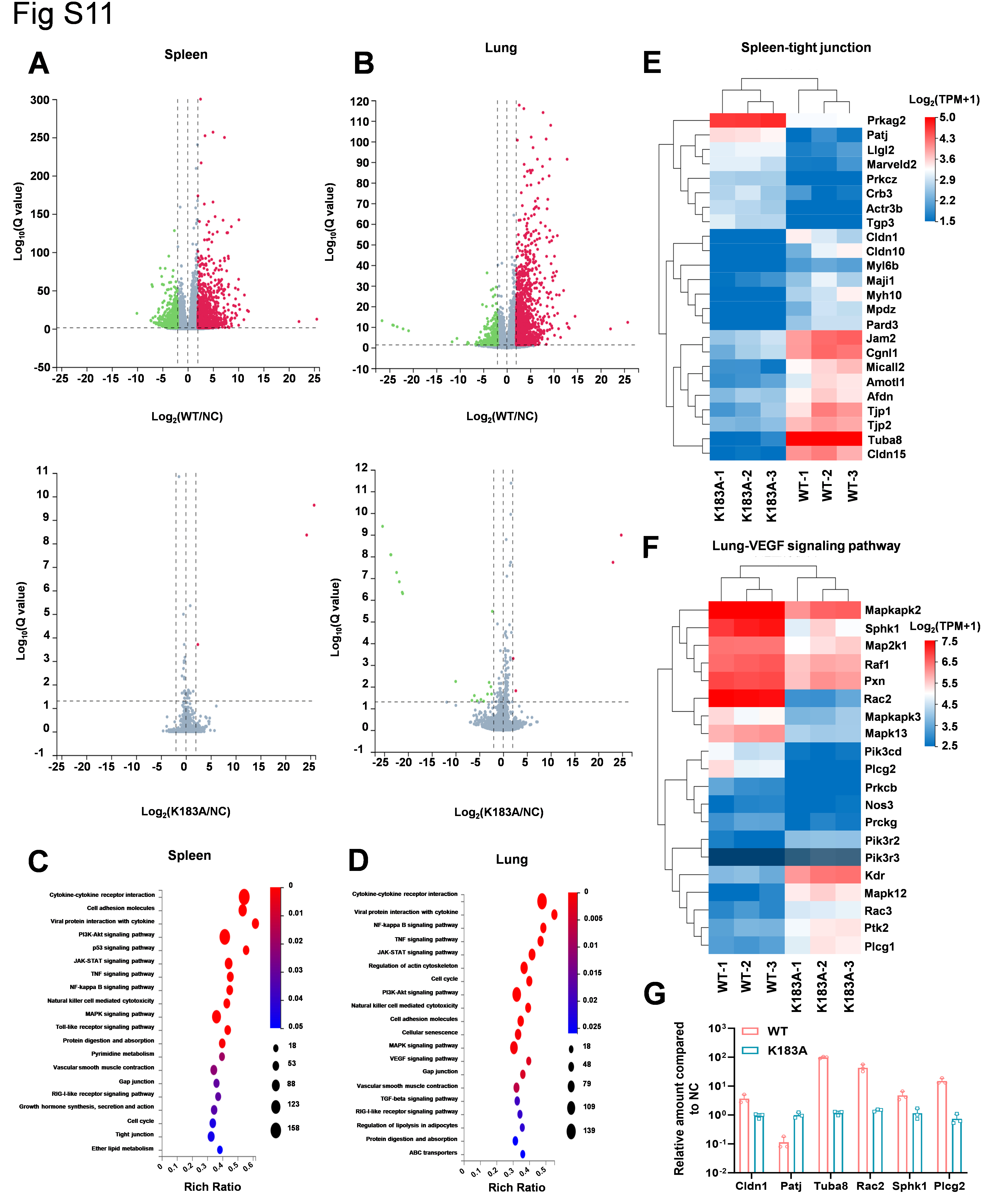


Figure S11. Differential Expression Analysis of RNA-seq Between BTV WT and BTV NS3_K183A_-Infected Mice. (A–B) Volcano plots of differentially expressed genes (DEGs) in spleen and lungs, with the log_2_ (fold change, FC) of gene expression, and the y-axis represents the log_10_ (Q value). Red points denote significantly upregulated genes (Q < 0.05, log_2_FC > 2), green points indicate significantly downregulated genes (Q < 0.05, log_2_FC < –2), and gray points represent non-significant changes. (C–D) KEGG pathway enrichment analysis of DEGs from (A–B) (P ≤ 0.05). (E–F) Heatmaps of DEGs in the tight junction pathway (spleen) and VEGF signaling pathway (lungs), with rows representing genes, columns representing samples, and color gradients indicating normalized expression levels (red = high, blue = low). (G) qPCR validation of RNA-seq-identified differentially expressed genes in spleen (Cldn1, Ptaj, Tuba8) and lung (Rac2, Sphk1, Plcg2) tissues. Primer sequences are provided in Table S4.

Figure S12


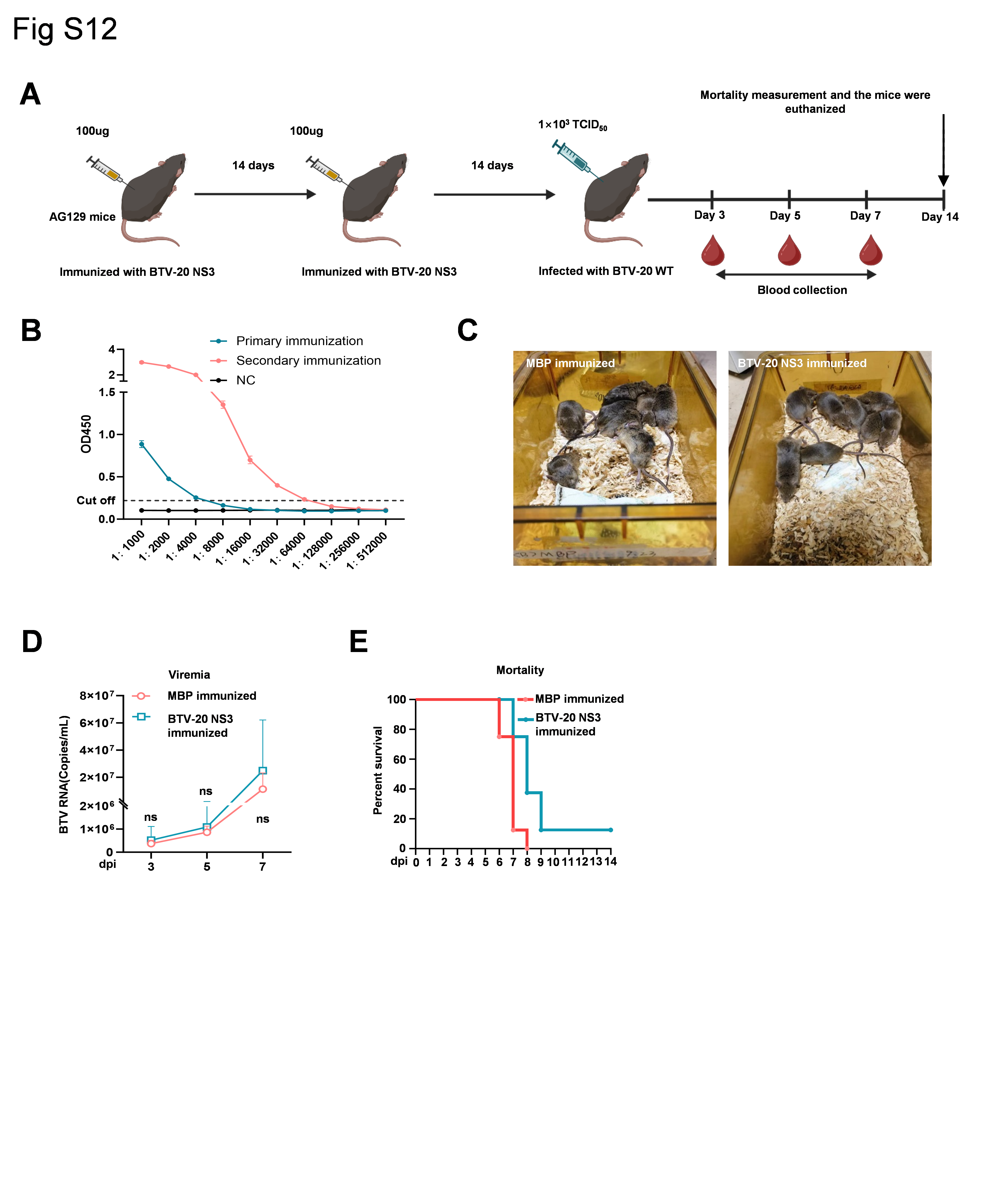


Figure S12. NS3 Immunization Delays Mortality and Confers Partial Protection from BTV Lethality in Mice. (A) Schematic representation of the NS3 immunization study design. AG129 mice received two doses of either MBP (control) or purified NS3 at 14-day intervals, followed by challenge with BTV WT (1×10^3^ TCID_50_). (B) Anti-NS3 antibody titers measured by ELISA. (C) Clinical presentation at 4 dpi. (D) Viremia levels quantified by qRT-PCR (n = 8). (E) Survival curves were recorded for 14 dpi (n = 8). The data shown in D are means ± SD. Statistical analysis was performed using two-way ANOVA, followed by Dunnett’s post hoc test for multiple comparisons. *P < 0.05, **P < 0.01, ***P < 0.001, ****P < 0.0001; ns, not significant.

Figure S13


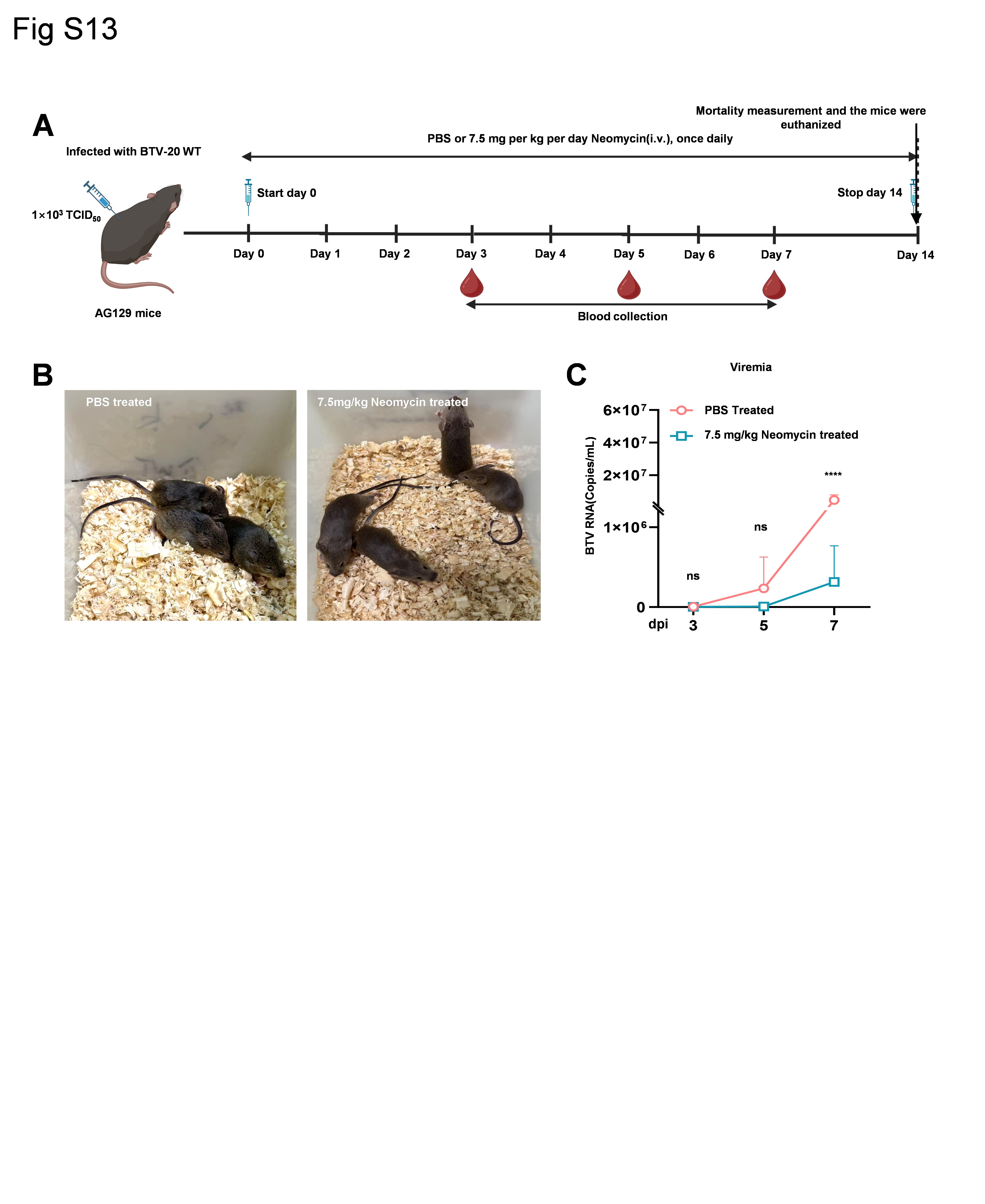


Figure S13. Neomycin Treatment Reduces BTV-induced Lethality in Mice. (A) Schematic representation of the Neomycin protection study design. AG129 mice were subcutaneously infected with BTV WT (1×10^3^ TCID_50_) and subsequently administered either PBS (vehicle control) or neomycin (7.5 mg/kg/day) via intravenous (i.v.) injection once daily. (B) Clinical manifestations at 5 dpi. (C) Viremia levels quantified by qRT-PCR (n = 5). The data shown in C are means ± SD. Statistical analysis was performed using two-way ANOVA, followed by Dunnett’s post hoc test for multiple comparisons. *P < 0.05, **P < 0.01, ***P < 0.001, ****P < 0.0001; ns, not significant.

| SiRNA | Sequence | SiRNA | Sequence |
| --- | --- | --- | --- |
| SAR1A-1 | CCGACAUCAGAAGAGCUAATT | SEC23B-3 | GAGAUGCUUGCUAAUCGAATT |
| SAR1A-2 | GGUGGACUGUGCAGAUCAUTT | SEC24A-1 | GGAAGUGUAACUUAUGUUATT |
| SAR1A-3 | GAUGCAAUCAGUGAAG AATT | SEC24A-2 | GGAUAUCCUUCACUUCAAATT |
| SAR1B-1 | GCGAGAGAUGUUUGGUUUATT | SEC24A-3 | GAGUGGAUUAAGUCUACAATT |
| SAR1B-2 | GGCACAGUACAUUGAUUAATT | SEC24B-1 | GGAGAUUCACGAACAAGAATT |
| SAR1B-3 | GGACAUGUUCAAGCUCGAATT | SEC24B-2 | GGUCAUUAUGCUAUGUCAATT |
| SEC13-1 | GGGAAGUAAAGAAGAUCAATT | SEC24B-3 | GGUGUUGACAGCUCUUCUATT |
| SEC13-2 | GACACGACUCCUCAGUGAATT | SEC24C-1 | CGAUGUACAUCCUAUAAUATT |
| SEC13-3 | CAUCAGACAGGUCCGUCAATT | SEC24C-2 | GGAGCUCAAGUCACUGUUATT |
| SEC16A-1 | GCGAGUUCUGGUUAUGCAATT | SEC24C-3 | GGCAGUUAUCACCAGCUUATT |
| SEC16A-2 | GGUUCUGGUGCUUCCGAAATT | SEC24D-1 | GCACUUGGAUAGACAACAATT |
| SEC16A-3 | CGAGUGACCAGUUCAGAUATT | SEC24D-2 | GCUGUGCUUUACACGACAATT |
| SEC16B-1 | CGAAGAUUACCGAGAGCAATT | SEC24D-3 | GCAGAGGAGGACAAGUUUATT |
| SEC16B-2 | CGCAAUCAGUUGUAUUCAATT | SEC31A-1 | GGCAGAUCCUGAAUUGUUATT |
| SEC16B-3 | GCAUGGAGGUUAUUCUUAATT | SEC31A-2 | CAGACUUUGUAGAGCACAATT |
| SEC23A-1 | GGUUGAUACUUGCAUGGAATT | SEC31A-3 | GACAGAAACAAGUUGACAATT |
| SEC23A-2 | CGACUGGAAGCUACAAGAATT | SEC31B-1 | GCCUGUGAUUGCUCAGAAATT |
| SEC23A-3 | GGAUGUGAAGGCAUUUCAATT | SEC31B-2 | AAGACUCCAGAAUGAAATT |
| SEC23B-1 | GGUGGUUGGAGAUGAAUUATT | SEC31B-3 | GCAGUGAGGUGGUAUAUAATT |
| SEC23B-2 | GGACCGUGUGUGUCAGAAATT | Scrambled | UUCUCCGAACGUGUCACGUTT |

**Table S1.** **Sequences of siRNAs targeting candidate genes.**

**Table S2.** **Primers and probes used for viral RNA quantification.**

| Primers name | Primers sequence (5’-3’) | Probe | Target genes |
| --- | --- | --- | --- |
| BTV-Fwd | TGGATAAAGCGATGTCAAA | FAM-AAGCTGCATTCGC  ATCGTACGC-BHQ | BTV-20  Segment 10 |
| BTV-Rev | ACATCATCACGAAACGCTTC |  |  |
| EHDV-Fwd | AAATTGCGCATGTCAGCTGCGGT | FAM-TTGCTCGCACCCG  GTGACGTGATCCA-BHQ | EHDV-7  Segment 9 |
| EHDV-Rev | CATCTGCATCTTTCTCATC |  |  |

**Table S3.** **Genbank accession numbers of orbivirus NS3.**

| Orbivirus | Genbank Accession Number |
| --- | --- |
| Bluetongue virus 20 | WEW07597.1 |
| Epizootic hemorrhagic disease virus 7 | QES86655.1 |
| African horse sickness virus 6 | AKP19850.1 |
| Equine encephalosis virus | AEP95960.1 |
| Yunnan virus | YP_443934.1 |
| Umatilla virus | YP_009047250.1 |
| Great Island virus | YP_003896068.1 |
| St Croix River virus | YP_052951.1 |
| Eubenangee virus | AFH41518.1 |
| Palyam virus | ALW83187.1 |
| Warrego virus | AIT55722.1 |
| Wallal virus | AIT55712.1 |
| Orungo virus | AFX73397.1 |
| Lebombo virus | AFX73386.1 |
| Chobar Gorge virus | YP_009158911.1 |
| Chenuda virus | YP_009158899.1 |
| Corriparta virus | AGT51064.1 |
| Wad Medani virus | YP_009158892.1 |
| Changuinola virus | AIV43215.1 |

**Table S4.** **Sequence-specific primers for relative quantitative PCR (qPCR) analysis.**

| Name | Sequence (5’-3’) | Target |
| --- | --- | --- |
| SAR1A- Fwd | ATCCAATGTGCCAATCCTTAT | Human SAR1A |
| SAR1A- Rev | CCGTAACCTTGCCTCTTG |  |
| SAR1B- Fwd | TGCTAATGTGCCTATACTG | Human SAR1B |
| SAR1B- Rev | CTTCTCCGTAACCTTGTC |  |
| SEC13- Fwd | CAGATGGACTACTATGGCAC | Human Sec13 |
| SEC13- Rev | CCCTGAGGTCGGCGATAAGGAT |  |
| Sec16A- Fwd | GCCGCCTACTACTACTACC | Human Sec16A |
| Sec16A- Rev | GACGCAGCACCATCATAG |  |
| SEC16B- Fwd | GGGGCCATGCTTTGTTCCTG | Human Sec16B |
| SEC16B- Rev | AACACGTGGCTGCCTGTGGA |  |
| SEC23A- Fwd | CTCAGTATCAGCATTCAAGT | Human Sec23A |
| SEC23A- Rev | ACATCTGGACCTTCTTCT |  |
| SEC23B- Fwd | ACTATGAGATGCTTGCTAAT | Human Sec23B |
| SEC23B- Rev | TTGGAATGTCTGCTTGAA |  |
| SEC24A- Fwd | TTGCTGTTGACTTATTCCT | Human Sec24A |
| SEC24A- Rev | GTTGTGCTGATGATGGTA |  |
| SEC24B- Fwd | GTCTCAGCCACTTGTTCATCTA | Human Sec24B |
| SEC24B- Rev | CAGAGCCACAGTCCATAAGG |  |
| SEC24C- Fwd | GTCTCCTCCTTCAGTCAG | Human Sec24C |
| SEC24C- Rev | AGCATCTCCATCTTGTCTT |  |
| SEC24D- Fwd | AAGGAAGAGCAAGAAGAGA | Human Sec24D |
| SEC24D- Rev | ATCAGTCACCACCATCAT |  |
| Sec31A- Fwd | GGAGGAAGCACAGATGGT | Human Sec31A |
| Sec31A- Rev | TCAGAGGCAGCACTATACTATG |  |
| SEC31B- Fwd | ATCCAGCCTTTGCCTTTGAG | Human Sec31B |
| SEC31B- Rev | TAGAGCCAGGTCGCATGATG |  |
| GAPDH- Fwd | GTCCATGCCATCACTGCCACCCAG | Human GAPDH |
| GAPDH- Rev | GCTGTTGAAGTCACAGGACACAAC |  |
| Cldn1- Fwd | ACTCCTTGCTGAATCTGAA | Mouse Cldn1 |
| Cldn1- Rev | AGCCATCCACATCTTCTG |  |
| Patj- Fwd | ATGGCTCTGGATTAGGTT | Mouse Patj |
| Patj- Rev | AACTTGTTCACTGGTCATC |  |
| Tuba8- Fwd | TACTTCTCTGCTGATGGA | Mouse Tuba8 |
| Tuba8- Rev | CGTAGATGGCTTCGTTAT |  |
| Rac2- Fwd | CCACTCTCCTATCCTCAA | Mouse Rac2 |
| Rac2- Rev | CCGTTCAATCGTATCCTT |  |
| Sphk1- Fwd | GTGAGGCTGGTGTTATGC | Mouse Sphk1 |
| Sphk1- Rev | CAGGTATGGACAGTCAAGTTC |  |
| Plcg2- Fwd | CCGCAGACAAGATTGAAG | Mouse Plcg2 |
| Plcg2- Rev | CCGTAGAGGATGGTGAAG |  |
| β-actin- Fwd | GCACCACACCTTCTACAA | Mouse β-actin |
| β-actin- Rev | TACGACCAGAGGCATACA |  |
